# The Robustness of Cellular Immunity Determines the Fate of SARS-CoV-2 Infection

**DOI:** 10.3389/fimmu.2022.904686

**Published:** 2022-06-27

**Authors:** Esther Moga, Elionor Lynton-Pons, Pere Domingo

**Affiliations:** ^1^ Department of Immunology, Hospital de la Santa Creu i Sant Pau, Biomedical Research Institute Sant Pau (IIB Sant Pau), Universitat Autònoma de Barcelona, Barcelona, Spain; ^2^ Unidad de enfermedades infecciosas, Hospital de la Santa Creu i Sant Pau, Barcelona, Spain

**Keywords:** SARS-CoV-2, cellular immunity, vaccine, COVID-19, helper T cells, cytotoxic T lymphocytes, severity, evasion

## Abstract

Two years after the appearance of the SARS-CoV-2 virus, the causal agent of the current global pandemic, it is time to analyze the evolution of the immune protection that infection and vaccination provide. Cellular immunity plays an important role in limiting disease severity and the resolution of infection. The early appearance, breadth and magnitude of SARS-CoV-2 specific T cell response has been correlated with disease severity and it has been thought that T cell responses may be sufficient to clear infection with minimal disease in COVID-19 patients with X-linked or autosomal recessive agammaglobulinemia. However, our knowledge of the phenotypic and functional diversity of CD8+ cytotoxic lymphocytes, CD4+ T helper cells, mucosal-associated invariant T (MAIT) cells and CD4+ T follicular helper (Tfh), which play a critical role in infection control as well as long-term protection, is still evolving. It has been described how CD8+ cytotoxic lymphocytes interrupt viral replication by secreting antiviral cytokines (IFN-γ and TNF-α) and directly killing infected cells, negatively correlating with stages of disease progression. In addition, CD4+ T helper cells have been reported to be key pieces, leading, coordinating and ultimately regulating antiviral immunity. For instance, in some more severe COVID-19 cases a dysregulated CD4+ T cell signature may contribute to the greater production of pro-inflammatory cytokines responsible for pathogenic inflammation. Here we discuss how cellular immunity is the axis around which the rest of the immune system components revolve, since it orchestrates and leads antiviral response by regulating the inflammatory cascade and, as a consequence, the innate immune system, as well as promoting a correct humoral response through CD4+ Tfh cells. This review also analyses the critical role of cellular immunity in modulating the development of high-affinity neutralizing antibodies and germinal center B cell differentiation in memory and long-lived antibody secreting cells. Finally, since there is currently a high percentage of vaccinated population and, in some cases, vaccine booster doses are even being administered in certain countries, we have also summarized newer approaches to long-lasting protective immunity and the cross-protection of cellular immune response against SARS-CoV-2.

## Highlights

. The presence of cross-reactivity, either humoral or cellular, between common cold hCoV and SARS-CoV-2 does not prevent infection but may be associated with less severe COVID-19.
. The presence of SARS-CoV-2-specific CD4+ Th1 IFN-γ-producing cells and CD8+ CTLs cells were associated with reduced disease severity.
. T lymphocyte recruitment to infected lung tissues and T lymphocyte apoptosis/necrosis caused by the cytokine storm might be crucial determinants of CD4+ and CD8+ T-cell lymphopenia in severe COVID-19 cases.
. Severe/fatal disease presents with excessive hyperactivation of immune function with increased Tregs and Th2 and/or Th17 cell-biased phenotype, leading to T cell exhaustion and subsequently to a state of anergy.
. Functional memory B and T cells to SARS-CoV-2 have been detected 12 months after natural infection. SARS-CoV-2-specific T cell memory may be long lasting given that COVID-19 convalescent patients develop SARS-CoV-2-specific TSCM cells that display a non-exhausted phenotype.
. The immunogenicity of SARS-CoV-2 vaccines involves the humoral response (number of spike-specific antibodies, neutralizing antibodies, and antibody neutralization capacity) and the cellular response (IFN-γ-producing CD4+ and CD8+ T cells). Therefore, a combined analysis of humoral and cellular immunity is necessary for the identification of vaccine responders and the immune protection evolution.

## Introduction

Coronaviruses (CoVs) are enveloped viruses containing non-segmented, single-stranded, positive-sense RNA genome whose primarily hosts are vertebrates (1, 2). Human Corovaniruses (HCoVs) have been responsible for significant health-related and economic costs worldwide for the last 20 years. The first time we received alarming information regarding HCoVs was with the appearance of severe acute respiratory syndrome coronavirus (SARS-CoV) in Guangdong Province (China) in November 2002 (3). By 2003, it had turned into a global infection with a mortality rate of 10% (4). The second HCoV outbreak occurred a decade later, in June 2012, with the Middle East respitatory syndrome coronavirus (MERS-CoV), which originated in Jeddah, Saudi Arabia (5). In the case of MERS-CoV, a 35% fatality rate was reported worldwide (6). Finally, the current pandemic, which is caused by severe acute respiratory syndrome coronavirus 2 (SARS-CoV-2), originated in Wuhan, China in December 2019 and causes the infection designated COVID-19 (Coronavirus Disease 2019) (7).

CoVs are classified in the realm Riboviria, order Nidovirales, suborder Cornidovirineae and family Coronaviridae with all 39 species of CoVs distributed in 27 subgenera, five genera, and two subfamilies (8, 9). HCoV are members of the Coronavirinae subfamily and are, in turn, categorized by the International Committee for the Taxonomy of Viruses into four major genera: AlphaCoV, BetaCoV, GammaCoV, and DeltaCoV (9). The AlphaCoV genera contains the common cold-causing HCoV-229E and HCoV-NL63 HCoVs, whereas in the BetaCoV genera are placed the common cold-causing HCoV-HKU1 and HCoV-OC43, as well as SARS-CoV, and MERS-CoV (10). SARS-CoV-2 sequence analysis has demonstrated a distant similarity of 79% to SARS-CoV and a 50% similarity to MERS-CoV with an 88% sequence identity to bat-SL-CoVZC45, bat-SLCoVZXC21 and bat-derived SARS-like CoV (11, 12).

The emergence of numerous SARS-CoV-2 variants of interest (VOI) and variants of concern (VOC) is one of the most important developments in the COVID-19 pandemic (13). The most important VOC variants reported to date are Alpha (B.1.1.7), Beta (B.1.351), Gamma (P.1) (14, 15), Mu (B.1.621) (16), Delta (B.1.617.2) (17) and Omicron (B.1.1.529), with the latest VOC reported in November 2021 (18). Understanding the impact of these variants on cellular immunity, in the context of COVID-19 infection and vaccination, is important for the development of effective strategies against future SARS-CoV-2 variants.

## Viral Infections and T Cell Immune Responses

A harmonized innate and adaptive immune response is crucial for the control and clearance of most viral infections. These two branches of the immune system collaborate to protect the body against infections. First, innate immunity includes evolutionarily primitive molecular and cellular mechanisms that recognize pathogens as common molecular patterns with the aim of preventing infection and quickly eliminating them. Second, the adaptive immune system takes longer to act but is characterised by a much more accurate response, as T and B lymphocytes undergo antigen-specific selection and proliferation. For many primary virus infections, it typically takes 7-10 days to prime and expand adaptive T cell immune responses in order to control the virus (19). Virus specific T cells have been shown to be protective against other viruses, like influenza (20), while heterologous immunity against diverse influenza strains is associated with conserved memory T-cell epitopes (21–23).

### Coronaviruses and Immunity: Previous Knowledge

The innate immune system includes physical and chemical barriers to infection, as well as the germline-encoded receptors, known as pattern recognition receptors (PRRs), which recognise the common molecular structures of many pathogens. PRRs bind to pathogen-associated molecular patterns (PAMPs) and damage-associated molecular patterns (DAMPs) and trigger cellular responses. As we know from other CoVs, double-stranded RNA (dsRNA), which is a by-product of viral genome replication and transcription, is a relevant PAMP model for CoVs (24, 25). It can be detected in the endosome by Toll-like receptor 3 (TLR3) and in the cytoplasm by RNA helicases retinoic acid-inducible gene I (RIG-I), melanoma differentiation-associated protein 5 (MDA5), and protein kinase R (PKR) (26–28). Single-stranded RNA (ssRNA) can also be detected in the endosome by Toll-like receptor 7 (TLR7) (29). Altogether, this allows for the detection of viral infection, activating signalling cascades like myeloid differentiation primary response 88 (MyD88) and inducing the production of type I interferons (IFNs) and nuclear factor kappa B (NF-κB) activation which, in turn, will induce the transcription of pro-inflammatory cytokines (30). Collectively, this triggers an antiviral immune response that constraints viral replication in infected and neighbouring cells.

However, CoVs are able to evade the mechanisms of innate immune detection, thereby preventing the generation of a proper immune response against viral infection (31). For example, the non-structural protein 3 (NSP3) of previous HCoVs has a papain-like protease domain that inhibits the activation of IFN regulatory factor 3 (IRF3) and the ubiquitination of TANK-binding kinase (TBK1), and RIG-I (32–35). Another example is the capacity of both SARS-CoV and MERS-CoV to prompt the production of double membrane vesicles lacking PRRs and their replication within them, thereby eluding the host viral dsRNA detection system (36, 37). Furthermore, the SARS-CoV and MERS-CoV M protein has previously been shown to interact with TNF receptor-associated factor 3 (TRAF3), disrupting TRAF3-TBK1 association and thus suppressing type I IFN production (38–40).

When the innate immune system is unable to control the viral infection, the adaptive immune system assumes a very important role. Previous studies of the adaptive immune response to earlier CoVs reported that antibody response decreases rapidly after infection or immunization, especially in cases of mild or subclinical disease such as that caused by common cold CoVs or mild MERS-CoVs, allowing for potential reinfection (41, 42). Moreover, SARS-CoV and MERS-CoV have been shown to impair T cell function and induce T cell apoptosis (43, 44). Thus, a commonly observed phenotype during acute phase disease in SARS-CoV and MERS-CoV patients, and also in COVID-19, was lymphopenia, which was seen particularly in patients with severe disease (43, 45, 46).

As is well known, the cytokine microenvironment generated by antigen presenting cells directs T cell phenotype differentiation and responses. Current evidence indicates that T helper 1 (Th1) response is crucial for the successful control of SARS-CoV and MERS-CoV (47).

There are a number of studies attempting to address the issue of the immune memory persistence conferred by infection with HCoVs. Some of those reveal that CD4+ and CD8+ memory T cell responses were identified in the blood of 70-100% of SARS-CoV patients four and six years after infection (48–50) and (51) even detected CD8+ T cell responses 11 years post-infection. These memory T cells may remain functionally active since another study revealed that they could proliferate, produce IFN-γ and induce delayed-type hypersensitivity (DTH) fast at antigen reencounter (48). Overall, T cell responses have been observed to have enhanced durability relative to antibody responses in SARS-CoV and MERS-CoV, hence it seems cellular response is crucial for the longevity of the immunity conferred by infection with CoVs.

### Pre-Existing Immune Reactivity

At the beginning of the pandemic outbreak, many studies focused on the possibility of pre-existing immunity against SARS-CoV-2. Considering that more than 90% of the human population is seropositive for at least one out of three of the common cold-causing HCoVs (52), it is reasonable to hypothesize that there may be a degree of cross-reactivity between the immunity conferred against common cold HCoVs and immunity against SARS-CoV-2. Among unexposed donors, 20% to 50% had lymphocytes exhibiting significant reactivity to antigen peptide pools of SARS-CoV-2 (53–57).

Multiple investigations into early serological response to SARS-CoV-2 reported unconventional seroconversion patterns resembling those of secondary immune responses. During a secondary immune response, memory lymphocytes provide the necessary mechanisms for rapid, antigen-specific, effective immune responses, and when the same pathogen infects the body a second time, it often originates only mild symptoms or may not cause any symptoms at all. A large serological study of COVID-19 patients found IgM seroconversion before IgG (typical primary response), as expected in previously unexposed individuals, but also synchronous IgM and IgG, and IgM after IgG seroconversion, describing an uncommon pattern of seroconversion to SARS-CoV-2 infection (58). In COVID-19 convalescent subjects, IgG against the S protein of the HCoV-OC43 had higher titers than in unexposed subjects but that was not true for the S protein of HCoV-229E, which suggests a more significant cross-reactivity between betacoronaviruses (59). The same authors suggested that the early parallel production of IgM and IgG in response to SARS-CoV-2 infection might be mediated by the stimulation of IgG memory B cells, as well as by naïve B cells (59) indicating that the memory generated by previous infections with other HCoVs would trigger a response to infection by the current SARS-CoV-2.

However, although there are studies supporting the presence of pre-existing SARS-CoV-2 cross-reactive antibody neutralizing capacity (60), others found no association between the presence of pre-existing cross-reactive antibodies to SARS-CoV-2 and protection against SARS-CoV-2 infections and hospitalizations (61). That might be explained by the fact that those pre-existing cross-reactive antibodies share predominantly non-neutralizing antibodies against the epitopes of previously circulating HCoVs (61–63). Assuming that shared cross-reactive antibodies to SARS-CoV-2 had no neutralizing activity, pre-existing cellular immunity would play a crucial protective role.

Several studies have provided evidence of the cross-reactivity of T cell responses between SARS-CoV-2 and the common cold HCoVs (55–57, 64–67). Mateus J. et al. (64) detected cross-reactive CD4+ memory T cells with peptide pools selected on the basis of homology between SARS-CoV-2 and other HCoVs and concluded that memory CD4+ T cells recognizing common cold HCoVs can exhibit substantial cross-reactivity to the homologous epitope in SARS-CoV-2. Cross-reactive CD8+ T cells also exist and, although they are less prevalent than cross-reactive CD4+ T cells (53), might be important determinants of immune protection at individual and population levels (68).

Nevertheless, pre-existing T cell immunity to SARS-CoV-2 has apparently low avidity when compared to that developed following infection with SARS-CoV-2 and may not participate in immunity very effectively (69). Thus, the immunity developed by previous HCoVs is not sufficient to prevent subsequent infection by SARS-CoV-2 but might be associated with less severe COVID-19 (70).

Interestingly, there seems to be an inverse association between cross-reactive antibody levels and age as shown by Shrwani K. et al. (63), who found children and younger people to have higher pre-existing cross-reactive antibodies to SARS-CoV-2 than older individuals. In line with that finding, a decrease in the magnitude and quality of SARS-CoV-2 cross-reactive CD4+ T-cells response with age has also been reported (71). Bearing this in mind, increased susceptibility to severe COVID-19 in elderly patients may at least in part be explained by a smaller pool of naïve T cells and the incapacity of the aged immune system to maintainthe SARS-CoV-2 cross-reactive T cells induced by previous HCoV infection.

## SARS-CoV-2 Immune Evasion

### Innate Immune System Evasion

As mentioned above, the first line of defense provided by our immune system against infection comes from the innate immune system. SARS-CoV-2, like other viruses and other HCoVs, attempts to evade the innate immune system and has been shown to do so by employing several different strategies.

Apparently, the main tactic by which viruses manage to evade the innate immune system is the inhibition of type I IFN response at different levels. It has been reported that SARS-CoV-2 may inhibit viral RNA recognition by modifying its own RNA and mimicking host RNA. The non-structural proteins NSP13, NSP14 and NSP16 perform this function by mediating the addition of a 7-methylguanyalte cap at the 5’ end of viral RNA in order to elude RIG-I and MDA5 recognition (72, 73). SARS-CoV-2 can also inhibit type I IFN at different points of the signalling cascade, leading to IFN production after non-self nucleic acid detection. The SARS-CoV-2 NSP15 protein may reduce IFN production as there is evidence that NSP15 binds to NRDP1 (74), the E3 protein ubiquitin ligase, which is known to enhance TBK1 and IRF3 activation, thereby promoting IFN production (75). TBK1 activation can also be inhibited by the NSP13 SARS-CoV-2 protein (74, 76, 77), and is decreased, along with IRF3 activation, by open reading frame 9 (ORF9)- cyclic GMP−AMP synthase (cGAS)-stimulator of interferon genes (STING) interaction (78). The NSP12 SARS-CoV-2 protein seems to impair the nuclear translocation of IFR3 by inhibiting IFN-β promoter activity (79, 80). Some studies have reported the disruption of RIG-I-like receptor (RLR) signalosome binding to translocase of outer mitochondrial membrane 70 (TOM70) by ORF9b (74, 76, 81) and have suggested that ORF9b-TOM70 interaction may inhibit IFN-β promoter activity (82). Moreover, ORF9b expression by SARS-CoV-2 may prevent the ubiquitination of NEMO (NF-kB essential modulator), NF-kB activation and nuclear translocation (83). Furthermore, the ISGlyation (labelling with interferon-stimulated gene 15 (ISG15), an ubiquitin-like protein) of MDA5, which is required for downstream pathway activation to lead to IFN-β secretion, may be inhibited by the NSP3 protein of SARS-CoV-2 (84). The NSP3 protein also seems to antagonize IRF3 stabilization (85). Other investigations have proposed that SARS-CoV-2-M protein antagonizes RLR signaling by inhibiting IFN-β and IFN-k gene expression and IFN-β promoter activity (86, 87). In addition, Xia H. et al. (88) demonstrated that M protein reduces ISRE (interferon-stimulated response element) reporter activity after treatment with IFN activation. SARS-CoV-2 has been also shown to inhibit the IFN signalling cascade at the signal transducer and activation of transcription (STATs) phosphorialtion level. For example, the expression inhibition and lysosomal degradation of interferon-α/β receptor 1 (IFNAR1) by NSP14 and ORF3a SARS-CoV-2 proteins impairs STAT1 phosphorylation, as reported by Hayn M. et al. (89).

### Adaptive Immune System Evasion

All the above-mentioned strategies allow SARS-CoV-2 to overcome the first line of defense of the host and this is when the host’s second line of defense comes into play: the adaptive immune system. Unfortunately, SARS-CoV-2 has also developed evasion mechanisms to overcome the adaptive immune system.

#### Humoral

A certain degree of SARS-CoV-2 antibody neutralization escape has been detected in every variant of concern: Alpha or B.1.1.7 (90, 91), Beta or B.1.315 (92, 93), Gamma or P.1 (94, 95), Epsilon encompassing the lineages B.1.427 and B.1.429 (96, 97), Delta or B.1.617.2 (17, 98), as well as Omicron or B.1.1.529 (99–101). As expected given the unprecedented high infection (and reinfection) rate numbers of the Omicron variant, this VOC was able to easily evade past infection humoral immunity compared to the epidemiological surveillance data for Beta and Delta variants (102). However, although the neutralization ability of convalescent sera against Omicron is low, a certain degree of neutralization still exists, indicating that there is still a certain level of protective effect (102). Another relevant mechanism used by SARS-CoV-2 to evade humoral response is the ability of this virus to spread from cell to cell without exposure to the extracellular environment (103). This reduces the likelihood of SARS-CoV-2 detection by SARS-CoV-2-specific antibodies and therefore limits the role of humoral immunity in preventing viral spread within the host.

#### Cellular

Notwithstanding these considerations regarding neutralization escape, T-cell immunity against SARS-CoV-2 seems to be more robust, since SARS-CoV-2-CD4+ and CD8+ T cell responses are not substantially affected by the Alpha, Beta, Gamma and Epsilon variants of concern (B.1.429), likely because T cell responses against SARS-CoV-2 are highly multi-antigenic and multi-specific, with many different epitopes being recognized by CD4+ and CD8+ T cells in a given individual (55, 67, 104–106). Nonetheless, a T-cell response reduction to SARS-CoV-2 variants of concern Alpha, Beta, and Gamma (107) has been demonstrated in vaccinated individuals and, as assessed in COVID-19 convalescent patients and vaccinated individuals, two SARS-CoV-2-spike mutations in the Delta (B.1.617.2) and the Delta plus (AY.2/B.1.617.2.2) may play a crucial role in HLA recognition and in reducing cellular immune response (108). In the case of vaccinated individuals, the above mentioned effect may be due to the fact that the multi-specificity conferred by natural SARS-CoV-2 infection cannot be achieved. Furthermore, SARS-CoV-2 is able to reduce T-cell response through a mechanism mediated by infected monocytes. These can directly reduce T cell response and inhibit epithelial cell survival through the hypoxia inducible factor 1-alpha (HIF-1a)/glycolysis-dependent axis, potentially contributing to immunopathology. This may explain why elevated glucose levels in diabetic individuals enhance viral replication and cytokine expression in monocytes (109).

Nevertheless, there are scarce studies investigating the T-cell immune escape of SARS-CoV-2 variants due to the difficulty of measuring T-cell response in clinical practice compared to antibody detection assays. More research needs to be conducted on this issue in order to draw firmer conclusions.

## Natural Infection With SARS-CoV-2 (COVID-19)

In natural infection, when innate immunity stimulates the adaptive response and sufficient effector T and B cells have proliferated and differentiated, they work together to rapidly and specifically eliminate infected cells and circulating virions. In an orchestrated immune response, the humoral branch alone cannot clear an ongoing infection and a cellular immune response will also be necessary. Thus, the presence of both T cells and antibodies is associated with the successful resolution of the average of cases of COVID-19 (53). T lymphocytes, the cells responsible for cell-mediated immunity, recognize the antigens present on antigen-presenting cells (APCs), and help phagocytes to destroy these microbes or to kill the infected cells. The best defined T lymphocytes are helper and cytotoxic (or cytolytic) T lymphocytes (CTLs), which present the cluster differentiation markers CD4+ and CD8+, respectively. T cells also assist B lymphocytes to proliferate and differentiate into plasma cells that secrete different classes of antibodies. This process requires a fairly well-defined time frame. In SARS-CoV-2 infection, following an incubation period of four to seven days before symptom onset, patients with COVID-19 progress towards recovery after seven to 10 days or else develop serious illness (110–112). The course of severe COVID-19 is characterised by an increased inflammatory response with a marked reduction in the number of T cells, frequently of both CD4+ and CD8+ T cells (113–116). In addition, symptomatic SARS-CoV-2 infection tends to elicit a higher peripheral blood T cell response with respect to asymptomatic infection (117, 118).The reduced frequencies of peripheral T cells during acute infection are likely to be associated with decreased CD4+ T cell proliferation and CD8+ T cell hyperactivation with T cell migration into the lungs (119). However, Liao L. et al. (120) have observed an increase of T cells in bronchoalveolar lavage fluids in mild patients but not in severe patients, suggesting a difference in T cell migration into the lungs in severe patients (120–122).

Meanwhile, arguments supporting the role of cellular immunity in the control of primary SARS-CoV-2 infection are supported by the fact that neutralizing antibody titers do not correlate with lessened disease severity in primary COVID-19 (123–125). Unlike neutralizing antibodies, SARS-CoV-2-specific CD4+ and CD8+ T cells were found to be associated with reduced disease severity in the same individuals (124). In agreement with these findings, there are reports of healthy individuals successfully controlling a SARS-CoV-2 infection with little to no neutralizing (or receptor binding domain -RBD- IgG) antibodies detectable post-infection, while having significant SARS-CoV-2-specific T cell memory (67, 68, 124, 126). On the other hand, neutralizing antibody titers (and total spike antibody titers) have indeed been positively correlated with COVID-19 disease severity (58, 127–129), possibly indicating that under normal conditions the adaptive immune response works in strict balance, but when one arm becomes unbalanced the other tries to compensate. Thus, a defect in the cellular response would cause a greater humoral response to correct this deficiency. The role of cellular response has also become evident in patients with agammaglobulinemia and no circulating B cells who have fully recovered from infection (130, 131) and subjects with pharmaceutical depletion of B cells who resolved COVID-19 infection without requiring intensive care (132–136). Moreover, in patients with haematological malignancy, CD8+ T cells appear to compensate for the lack of humoral immunity and were associated with improved outcomes, indicating a role for T cells in protection against SARS-CoV-2 infection (137).

**Figure 1:**
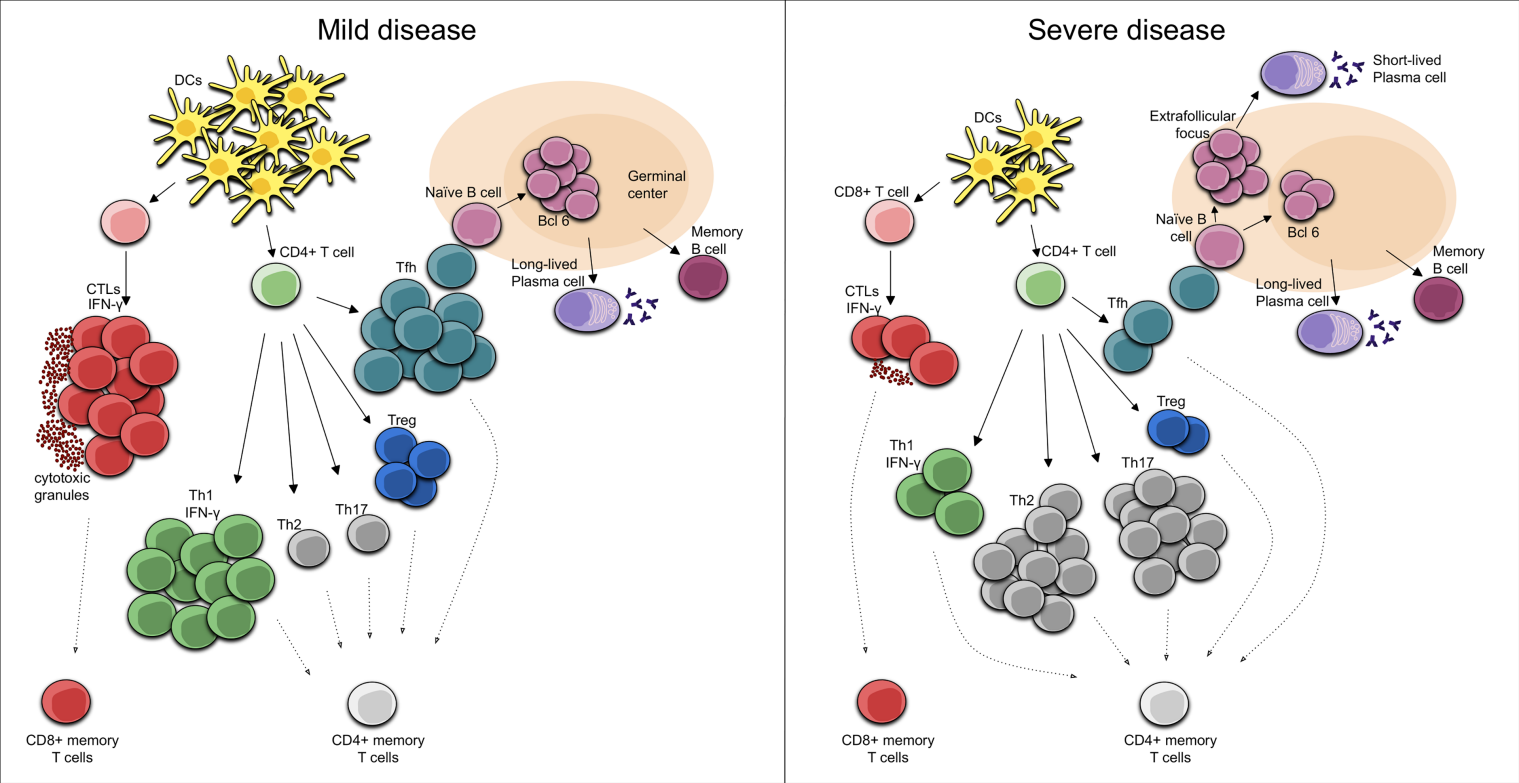
Cellular immune response in Mild COVID-19. In mild COVID-19, there is an early induction of the Th1 cell-biased phenotype with IFN-γ secreting SARS-CoV-2-specific T cells. In turn, SARS-CoV-2-specific CD8+ T cells perform rapid responses, acting as CTLs, secreting cytotoxic granules and high levels of IFN-γ. Moreover, activated Tfh cells in the draining lymph nodes activate the naïve B cells that are necessary for the development of long-lived plasma cells and memory B cells. Cellular immune response in Severe COVID-19. During a severe course of COVID-19, there are reduced numbers and functions of DCs, leading to decreased numbers of CD4+ T cells. In this case, an elevation of Th2 phenotype and/or a dysregulation of the Treg/Th17 cell ratio toward the Th17 phenotype can be seen. Furthermore, decreased numbers of CD8+ T cells with an exhausted phenotype results in reduced CTL functionality while the T cell-mediated activation of B cells in extrafollicular focus induces their differentiation into short-lived plasma cells.

CD4+ T cell responses to pathogens are divided into three major types: Th1, Th2, and Th17. Th1 immune response, which is characterized by T-bet-dependent responses and IFN-γ secretion, is generated against intracellular pathogens including viruses. In the Th1 response, pathogen clearance is mediated through effector cells including innate lymphoid cells 1 (ILC1), NK cells, and cytotoxic T lymphocytes (138–140). During SARS-CoV-2 acute infection, patients display a proliferation of IFN-γ-producing Th1 (IFN-γ, IL-12, IL-15, IL-2 and TNF) cells and it has been suggested that this Th1 cell-biased phenotype is associated with less severe disease (54, 141). In patients with moderate disease, the core COVID-19 inflammatory cytokine signature with IL-1a, IL-1b, IL-17A, and IFN-α observed in the first 10 days from symptom onset declined steadily (142) and the same happens with the innate cytokine IL-12, a key inducer of Th1 immune response, as well as IFN-γ (142). Early induction of IFN-γ-secreting SARS-CoV-2-specific T cells with accelerated viral clearance is present in these patients with mild disease (125) **Figure 1**.

The effector cells of CD8+ lineage are CTLs, whose major function is to eliminate cells harboring viruses. SARS-CoV-2 CD8+ T cells are specific for a range of SARS-CoV-2 antigens, and spike, nucleocapsid, M, and ORF3a proteins are well represented (53, 56, 67, 126, 143, 144). CD8+ CTLs eliminate intracellular microbes mainly by killing infected cells by releasing cytotoxic proteins stored within cytoplasmic granules to the target cell and subsequently triggering cellular apoptosis. In acute COVID-19, SARS-CoV-2-specific CD8+ T cells exhibit high levels of IFN-γ, granzyme B, perforin, and CD107a molecules, some of which are present in the cytotoxic granules and are associated with potent cytotoxic effector functions (68, 124, 126, 145), developing fast CD8+ T cell responses (124). Patients with milder disease and recovery have been associated with a more robust clonal expansion of CD8+ T cells in peripheral blood (146). These findings would explain why, in SARS-CoV-2 infections, the presence of virus-specific CD8+ T cells has been associated with better COVID-19 outcomes (124, 144). Taken together, these observations suggest that cytotoxic activity is critical for the clearance of many viral infections and is therefore also important for the eradication of the infection reservoir. As with peripheral blood results, there is also an increase in SARS-CoV-2-specific CD8+ T cells in the respiratory tract of moderate COVID-19, as demonstrated in bronchoalveolar lavage fluid collected from COVID-19 patients. Bronchoalveolar lavage fluid CD8+ T cells showed clonal expansion, suggesting T cell migration to the infected site resulting in the overall peripheral counts (147).

The role of T folicular helper (Tfh) cells at germinal centers in the development of a long-lasting, high-affinity antibody response is well known (148, 149). In T cell-dependent immune responses, T cells are important in the formation of an extrafollicular focus, in which B cells proliferate and differentiate into plasma cells, most of which are short-lived. The activation of T cells in the extrafollicular focus will cause some of them to develop into Tfh cells and migrate into the germinal centers, where they perform their functions, which are necessary for the development of both the bone marrow resident plasma cells and the memory B cells that enter in the recirculating lymphocyte pool (150, 151). Importantly, it appears that the germinal center reaction in humans after vaccination persists over a longer period (152–154). Evidence suggesting that the above described process is indeed what occurs following SARS-CoV-2 infection have been provided by Mudd PA et al. (155) given that a high-magnitude, SARS-CoV-2-specific CD4+ T cell response in the draining lymph nodes is present during the development of high-titer neutralizing antibody responses in the setting of COVID-19 mRNA vaccination. The fact that CD4+ T cells in this type of response provide help to B cells for the production of antibodies has been demonstrated in other situations. For example, individuals with uncontrolled HIV and extremely low CD4+ T cell counts during vaccination lack seroconversion (156), and this has also been observed in patients subjected to T cell-focused immunosuppressive regimens following solid organ transplantation who received a standard two-dose BNT162b2 regimen (157). Overall, there is direct and indirect evidence of the need for a robust T response for the generation of high-titer neutralizing antibody responses following COVID-19 infection or mRNA vaccination. However, a lower quality and lack of durability of humoral response has been observed during natural SARS-CoV-2 infection. Thus, although there is evidence of a robust T-cell-mediated activation of B cells in the non-germinal-center, this may be due to a loss of germinal centers through a specific block of germinal center type B cell-lymphoma 6 (Bcl-6)+ T follicular helper cell differentiation (158) **Figure 1**. This may compromise the early development of the high-affinity antibodies that could contribute to a certain attenuation of viral spread. Moreover, in COVID-19 patients, the relationship between plasmablasts and activated Tfh is weak, even though these individuals have a robust plasmablast response (114). At least part of the plasmablast response may be through activated (CD38+HLA-DR+) CD4+ T cells, which might play a role in providing B cell help as a part of an extrafollicular response (114).

### Relation of Cellular Components With Disease Severity

As mentioned above, an immune response properly coordinated in time between the different components of innate and adaptive immunity is essential for it to be successful. In fact, if the adaptive immune response starts too late, fatal COVID-19 develops, defined as a situation in which the viral load is high (159).

IFN-γ has already been discussed as a cytokine secreted by CD4+ Th1 cells, but it is also secreted by differentiated CTLs. It contributes to classical macrophage activation and inflammation in the host’s defense and in hypersensitivity reactions. It is likely that both CD4+ Th1 cells and CD8+ T cells contribute to the IFN-γ–induced phagocytic clearance of ingested microbes. These functions would explain the beneficial effect of rapid IFN-γ secretion in response to an infectious process. Thus, Zheng M. et al. (160) reported the secretion of IFN-γ by both CD8+ T cells and CD4+ Th1 cells under conditions of severe COVID-19 disease. Therefore, a poor T cell response contributes to SARS-CoV-2 viral persistence and COVID-19 mortality, whereas strong T cell responses are protective in the majority of individuals. As seen in SARS-CoV-2 infection in non-human primate models, the deletion of CD8+ T cells impairs this protection (161).Thus, human individuals with higher levels of IFN-γ secreting T cells (measured by enzyme-linked immunosorbent spot assay) against the SARS-CoV-2 S protein, nuclear proteins, and membrane proteins have a better protection against the virus (162), while a CD4+ T cell IFN-γ expression decrease has been reported in severe SARS-CoV-2 infection peripheral blood samples, and the T cells of these patients seemed to be unable to produce IFN-γ in response to viral proteins (163).

Patients with severe COVID-19 have marked reductions in the number and frequency of both CD4+ and CD8+ T cells, but increased activation of T cells (142, 145). Specifically, in an autopsy report, low levels of hyperactive T-cells in peripheral blood and an accumulation of mononuclear cells in the lungs of the individual were observed (164). In addition, the high proportion of M/NP-specific CD8+ T cell responses compared to the spike-specific CD4+ T cell response seen in mild disease is not found in severe disease (144). In fact, the percentages and absolute numbers of CD8+ T cells in severe disease were significantly reduced (163). This finding could suggest a protective role of CD8+ T-cell response in mild disease or a pathogenic role of the CD4+ T-cell response in severe disease (144). The same applies in the case of ICU (intensive care unit) patients: total T-cell, CD4+ and CD8+ T-cell counts in peripheral blood were significantly lower than in non-ICU COVID-19 cases, and the counts correlated negatively with patient survival (165). Some authors have observed an increased expression of the inhibitory receptor NKG2A, suggesting a decrease in CD8+ T cell functionality (160). NKG2A expression is upregulated on NK cells and CTLs in COVID-19 patients, with a decreased capacity to produce CD107a, IFN-γ, IL-2, granzyme B and TNF-α., which suggests functional exhaustion of cytotoxic lymphocytes in COVID-19 patients (160). The upregulation of NKG2A expression may be a consequence of and to compensate for the hyperactivation of CD8+ T cells in the severe stage of COVID-19.

There is a cellular subset composed mainly of mucosal-associated invariant T (MAIT) cells, the CD161+ CD8+ T cells which undergoes a strong reduction in frequency in individuals with severe COVID-19 (145). During viral infections, MAIT cells can become activated and migrate to infection sites (166, 167). The sharp decline in circulating MAIT cells in severe COVID-19 patients correlates with their presence in the airways of the patients (168). The reduction of this population in peripheral blood is likely to be indicative of sequestration in the lungs, potentially exacerbating tissue inflammation.

During many acute viral infections, the period of peak T-cell responses and plasmablast detection in peripheral blood is relatively short (169–171). However, there is a subgroup of COVID-19 disease patients with an over-aggressive immune response and/or a “cytokine storm” (172) due perhaps to a failure to regulate responses or a prolonged period of peak immune responses because there is a stability over time of CD8+ and CD4+ T-cell activation and plasmablast response (114). There has been speculation on possible causes for the well-known cytopenia occurring in COVID-19. One of these causes may be related to the recruitment of T cells to infected lung tissues to control viral infection (173). Another cause might be the apoptosis or necrosis of T cells caused by the cytokine storm that occurs in severe cases of COVID-19 (174). The severity of the disease also correlates with cytokine levels and these patients secrete higher levels of IL-6 and IL-10 (175). Thus, in ICU patients a further increase in IL-6 and IL-10 plus TNF-α (165) has been observed and has also been found to be higher in the bronchoalveolar lavage fluid of deceased patients than in those who survive IL-6 (120). The increase in TNF-α may explain why antibody levels correlate with disease severity, since this cytokine secreted by CD4+ T cells serves as a co-stimulatory signal for B cells. Furthermore, the expansion of plasma cells in severe disease has been associated with large and oligoclonal B cell expansions (145).

The significant lymphopenia that COVID-19 patients present in the acute and severe phase is associated with a lower number and the functional impairment of dendritic cells (DCs), which are fundamental in T-cell antigenic presentation, compared to mild patients (119, 145, 176), and those cells are significantly decreased in fatal cases compared to survivors (177). Plasmacytoid DCs (pDCs), which is responsible for the production of the type I IFNs involved in virus defense, were also mainly reduced in abundance and impaired in function in severe COVID-19 patients (145). In fact, during COVID-19 infection, the rapid loss of DCs numbers and function may contribute to delayed T cell responses and the features of low level IFN-I/IFN-III (178).

This would partially explain the correlation of a fatal disease course with the age of the patients since we should not forget the process known as “immunosenescence”, which features a reduction in the ability to fight novel infection (179) and a reduced abundance of DCs in elderly patients (180). Thus, the presence of an immunosenescent phenotype, demonstrated by an elevated neutrophils-to-lymphocytes ratio, was found in severe COVID-19 patients but not in mild disease (181).

Furthermore, patients with severe fatal disease up to 10 days from the onset of symptoms have a excessive hyperactivation of the immune function, demonstrated by significantly increased HLA-DR expression and IFN-γ synthesis. In fact, a robust T cell response in critical patients may contribute to hyperreactivity and immunophatogenesis (182). Moreover, the proportion of T regulatory (Treg) lymphocytes increases significantly in this phase, which negatively regulates immune response (112). As discussed previously, the Th1 cell-biased phenotype is associated with less severe disease (54, 141), but patients with SARS-CoV-2-induced acute respiratory distress syndrome (ARDS) often tend to have a Th1:Th2 ratio weighted towards the Th2 type, leading to substantial lung tissue damage (183, 184). In addition, a broad elevation of Th1, Th2 and Th17 signatures, including inflammasome-dependent cytokines such as IL-1b, IL-18 and Th2 and Th17 cytokines has been identified in patients with severe COVID-19 (142). Th2 and Th17 immunity depend on the transcription factors GATA-3 and RORγt, respectively, and the predominant response is driven by (IL-4, IL-5, IL-13) and (IL-17, IL-22) respectively (138–140). Also, dysregulation of the Treg/Th17 cell ratio toward the Th17 phenotype is an important contributor to disease severity. IL-17 secreted during SARS-CoV-2 infection can promote migration of neutrophils and monocytes into the pulmonary interstitium resulting in its consequent inflammation, as well as the activation of other cytokine cascades (G-CSF, TNFα, IL-1β and IL-6), which contribute to aggravating this inflammation and tissue damage (185). Thus, patients with severe COVID-19 showed a markedly high number of CCR6+ Th17 cells in peripheral blood (164), even though not all patients with severe COVID-19 have increased IL-17 expression (163). These data suggest that the dysregulation of Th polarization occurs in severe COVID-19 and a bias towards this type of Th response might define the disease course **Figure 1**.

Whether or not infection and hyperactivation persist, the immune system eventually enters an anergy state in which the number of lymphocytes (including T and B lymphocytes), NK cells and DCs continues decreasing in patients with a fatal outcome. CD4+ T cell function is impaired, as evidenced by decreased activating receptors and an increased expression of CD45RA and CD28 (112). Thus, deceased patients have lower frequencies of HLA-DR+ and IFN-γ-secreting cells within CD4+ and CD8+ T cells than survivors (186, 187).

Another important factor to be taken into account during any maintained immune response is a phenomenon called exhaustion, which is observed, for example, in some chronic viral infections when CTL effector responses gradually extinguish over time (188). Exhausted cells express increased levels of multiple inhibitory receptors, notably programmed cell death-1 (PD-1), since the programmed cell death-ligand 1 (PD-L1)/PD-1 immune checkpoint axis is the strongest T cell exhaustion inducer, alongside cytotoxic T lymphocyte-associated protein 4 (CTLA-4), T cell immunoglobulin mucin-3 (TIM-3), lymphocyte activation gene-3 (LAG-3), and others. It has been reported that increased T cell exhaustion, observed by the high level expression of PD-1 and TIM-3 (165) induced by IL-10 (189) and decreased functional diversity correlates with the degree of disease severity in patients with COVID-19 (190). In particular, Kreutmair S. et al. (191) showed that CD4+ T cells increased PD-1 expression during the first days following hospital admission and then normalized in moderate patients but remained elevated in severe disease (191). Likewise, as in memory CD4+ T cells, the frequency of PD-1 expressing cells were reported to be higher after one month in recovered patients with severe COVID-19, and correlated with the age of the patient (145). However, Rha M.S. et al. (192) reported that SARS-CoV-2-specific CD8+ T cells expressing PD-1 were found not to be exhausted but functional. This is explained by the fact that PD-1 is expressed on exhausted T cells but is also expressed on recently activated T cells (193–196) and the persistence of antigen encounter results in the maintenance of PD-1 expression, leading to exhausted T cells (197). PD-1 expression in the peripheral blood of COVID-19 patients is also increased in the exhaustion of other T cell subsets such as γδ T, mucosa-associated invariant T and invariant NKT cells which, in agreement with their exhausted phenotype, produce less IFN-γ than cells from healthy donors (168). Also, the T cells of ICU patients expressed increased PD-1 in bronchoalveolar lavage fluid as compared to peripheral blood T cells (198).

Regarding ligand PD-L1, both soluble and membrane-bound PD-L1 increased levels are associated with the degree of severity in COVID-19 (199–201). PD-L1/PD-1 overexpression in the white adipose tissue of obese individuals during IFN-γ secretion, which leads to the dysfunction of T cells and especially to a reduction in cytotoxic activity, explains why SARS-CoV-2 infection can worsen disease in obese individuals (202). Overall, we can outline that T cells of COVID-19 patients display a higher expression of PD-1 and that this elevated expression is correlated with disease severity, but whether or not PD-1 expressing T cells in COVID-19 are functional needs to be investigated further. To assess exhaustion, it will be important to take into account not only the expression of PD-1, but other exhaustion markers and the time since a particular cell has encountered the antigen in order to differentiate an exhausted cell from a recently activated cell.

In the resolution of inflammation when the virus is eliminated, both adaptive regulatory cells, such as regulatory T and B cells and innate immune cells, such as macrophages and regulatory DCs, also contribute (203). In recovered patients, the number of peripheral blood lymphocytes gradually increases (186, 187) with a marked high frequency of spike specific CD4+ T cell response (53, 126, 144), while the effector function of T cells is not compromised (204). Two to four months after SARS-CoV-2 infection resolution, most of the components of cellular immunity return to normality (204), though with significant increases in regulatory T cell frequencies and TIM-3 expression on CD4+ and CD8+ T cells, while the cytotoxicity of T cells is significantly diminished (204). However, this immune response reversion is slower and the virus clearance time is prolonged in some critically ill patients even after entering the recovery stage (112).

## Balance Between Innate and Adaptive Immunity

The first contact with pathogens is established by the host innate immune system. It is noteworthy that the innate immune system is indeed capable of eliminating some infections on its own, particularly when the infection is localized and caused by a low number of pathogens. But innate immunity is not sufficient to protect us fully from infectious diseases, in part because, as discussed earlier, many pathogens have features that allow them to evade innate immune responses. At this early stage, the innate cytokine IL-12 has been shown to stimulate the differentiation of naive CD8+ T cells into effector CTLs and it is involved in the differentiation of CD4+ T cells into Th1 cells, both contributing to the IFN-γ–induced phagocytic clearance of ingested microbes. However, in some circumstances, the innate immune response seeks to fill the gap left by the absence of a T cell response, attempting to assume t control of the immune response against the virus with an ever-expanding innate immunity activation. Following this thread, there are many studies that have identified innate cytokine/chemokine signatures of immunopathology (145, 205–209). The most common observation in this line is an elevated frequency of neutrophils in blood (145) and massive numbers of neutrophils in the lungs, both of which are associated with severe, end-stage COVID-19 disease (147, 206–208), as well as the cytokine storm (172). In severe COVID-19 patients, IL-12 and IFN-γ increased over time; however, T cell depletion was detected in these patients and the remaining T cells did not produce larger amounts of IFN-γ (142). This suggests that the secretion of IFN-γ by innate cells, such as ILCs and NK cells, or resident T cells in tissues were the primary contributors to the enhancement of the IL-12 and IFN-γ cytokine levels observed in severe patients.

The other finding reported is the role that the sex of the patient plays in the type of predominant immune response. It has been shown that male patients have higher plasma levels of innate immune cytokines, including IL-8 and IL-18, along with activated non-classical monocytes. In contrast, female patients seem to generate a more robust T cell activation during SARS-CoV-2 infection. A poor T cell response might be responsible for the worse outcomes observed in male patients, while in female patients, higher levels of cytokines related to innate immune response appear to be associated with worse disease evolution (210).

A recently published study in mice suggests that specific T cell and antibody responses develop independently of SARS-CoV-2 detection by some of the pattern recognition receptors (PRRs) of the innate immunity system: TLR2-5 and TLR7, STING-cGAS, NLRP3 (inflammasome activation), as well as RIP3 kinase (mediator of nedroptosis) and gasdermin D (mediator of pyroptosis). On the other hand, these specific T cell responses, mainly featuring CD8+ T cells, are affected by the altered recognition of SARS-CoV-2 by the MDA5-IFNAR1 signalling pathways (211). Airway epithelial cells from children appear to show an increased expression of MDA5 compared to its expression level in SARS-CoV-2 positive adult epithelial cells (212). Consistent with this, we found several studies showing that children eliminate SARS-CoV-2 faster than adults, probably by detaining viral replication earlier (213–216).

In general terms, we have sought to emphasize that a balance between the innate and the adaptive immune response is paramount for a favourable evolution and resolution of COVID-19 disease and its imbalance has detrimental consequences, including the inability to configure a competent adaptive response or the overactivation of the innate immune system which results in a cytokine storm.

## Immune Memory

The balance between naïve and memory T cells is crucial for infection control. Naïve T cells are responsible for primary infection response and memory T cells promote antigen-specific immune responses, being able to protect the host from re-infection with the same pathogen. Immune memory against SARS-CoV-2 correlates positively with patient disease severity during acute phase infection, both in humoral and cellular response (217). Thus, it has been shown that memory B cells percentages among hospitalized cases were significantly higher than among non-hospitalized cases following infection (218).

There are some studies that report relatively stable humoral immunity for up to 6-12 moths post-infection (217–221) and Zhang J. et al. (217) described the detection of neutralizing antibodies in convalescent COVID-19 patients even at 12 months following symptoms onset. However, further studies show a clear decline of SARS-CoV-2 neutralizing antibodies in the first months after infection (222–224), along with a progressive decline in total antibody levels eight months after SARS-CoV-2 infection (218, 225). These inconsistencies in the results of humoral immunity longevity may be due to variations between the studied cohorts and the use of different techniques or distinct antibody-detection epitopes in the assays.

In a longitudinal study, Rodda L.B. et al. (226) detected memory T cells, which secrete IFN-γ and are able to clonally expand following SARS-CoV-2-antigen re-exposure, at least three months after disease onset. Further investigations have detected maintained SARS-CoV-2-specific memory T cell responses in COVID-19 convalescent patients at least 7-12 months after infection (217, 221, 227) and this has been found to be true regardless of disease severity (227). Considering immune memory at the tissue level, SARS-CoV-2-specific lung resident memory T cell can be detected at least 10 months following infection (228). Lung resident memory T cells may be key players in limiting the severity of COVID-19 or the potential for reinfection. In this regard, it has been described that a higher number of these resident memory T cells in the lungs corresponds with a higher degree of clinical protection (229).

Therefore, both B and T memory cells exhibit robust memory response (225), indicating that, in the event of a re-encounter with SARS-CoV-2, the levels of total and neutralizing antibodies and effector T cells necessary to respond efficiently to infection might be rapidly recovered. In fact, B and T cell memory functional responses to SARS-CoV-2 are still detectable 12 months after natural infection (230).

### Immune Memory Phenotypes

Individuals who have undergone mild symptomatic SARS-CoV-2 infection show, after a few months, an increase in circulating Th1 cytokine-producing CXCR5+ Tfh and CXCR5- non-Tfh cells, CD4+ CXCR3+ proliferative memory T cells and IFN-γ-producing CD8+ T cells (226). In these mild COVID-19 cases, CD8+ T memory cell responses predominate over CD4+ T memory cell responses and, additionally, the memory CD8+ T cells specific for SARS-CoV-2 M and NP proteins exhibit the highest frequency of multiple cytokine production (144). Additionally, the SARS-CoV-2-specific memory CD4+ T cells of recovered individuals have the capacity to express CXCR5, ICOS, CD40L and proliferate at spike-protein re-exposure (226). The expression of these markers and a variety of cytokines is important for T-B cells interaction (231) as they enable memory CD4+ T cells to help reactivate memory B cells and therefore start producing antibodies against SARS-CoV-2 since, as discussed above, their levels may have decreased over time. This may serve to explain why, during the memory phase, an abundance of Tfh cells correlates with antibody response (232–234).

Regarding the immune memory to SARS-CoV-2 in convalescent individuals, a number of authors have underlined the contribution of a subtype of terminally differentiated memory cells: the terminally differentiated effector memory T cells re-expressing marker CD45-RA (T_EMRA_). T_EMRA_ have generally been associated with protection against viral infection (22, 235–238). During the memory phase following SARS-CoV-2 infection, a high prevalence (218, 227) and a progressive enrichment of the T_EMRA_ phenotype and T stem cell memory (T_SCM_) phenotype in SARS-CoV-2-specific CD8+ T cells (239) has been described. The same authors postulate that the differentiation towards one phenotype or the other might be associated with disease severity with a bias towards T_SCM_ in mild disease and increased T_EMRA_ in severe disease. In agreement with previous studies that highlight the role of type I IFN in memory development (240), Adamo S. et al. (239) reported an expression enrichment of the genes involved in IFN signaling pathways in SARS-CoV2-specific memory CD8+ T cells. Thus, type I IFN signaling might be a key driver directing cells to become long-lived memory cells. While it has already been mentioned that T_EMRA_ cells are associated with protection against viruses, it has also been shown that they can accumulate during chronic viral infections (241). In Long COVID syndrome, when compared to COVID-19 convalescent individuals, an increase in CD8+ T effector memory (T_EM_) and CD8+ T_EMRA_ cell number, accompanied by a decrease in their functional activity, has been reported (242).

The success of long-term memory T cells depends on the generation of T_SCM_ cells (243) since they have a higher self-renewal ability and are multipotent cells, being able to reconstitute several memory phenotypes (244). T cell memory developed during SARS-CoV-2 infection may be long-lasting since COVID-19 convalescent patients develop SARS-CoV-2-specific T_SCM_ cells (192, 227, 239). Cohen K.W. et al. (245) defined most SARS-CoV-2 CD4+ T cells as displaying a central memory profile. Furthermore, Gurevich M. et al. (221) reported the presence of IL-2-secreting and IFN-γ+IL-2-secreting SARS-CoV-2-specific central memory T cells that might be long-lasting memory phenotypes in accordance with previous studies (246). There are two different subsets of CCR7+ stem cell-like progenitors: CCR7+PD-1−TIGIT− cells are observed to display stem cell-like features, whereas CCR7+PD-1+TIGIT+ cells seem to exhibit exhausted traits (192, 247). SARS-CoV-2-specific T_SCM_ cells rarely express PD-1 and TIGIT, making them non-exhausted-like progenitors but functional memory T cells (227).

## Vaccination

A good adaptive immune response and immune memory are vital to the success of vaccines and the achievement of a low degree of reinfection. When studying natural immunity to the virus, including the role of SARS-CoV-2 specific T cells, it is critical to fill in the current gaps in our knowledge for improved vaccine design. The generation of a robust cellular immune response is a desirable attribute for a vaccine against SARS-CoV-2 because, as we have referred to throughout this report, following natural infection, t T-cell response is activated rapidly to control disease progression (53, 55, 144, 248), and these virus-specific T-cell response have been shown to be associated with milder disease in COVID-19 patients (126).

**Figure 2:**
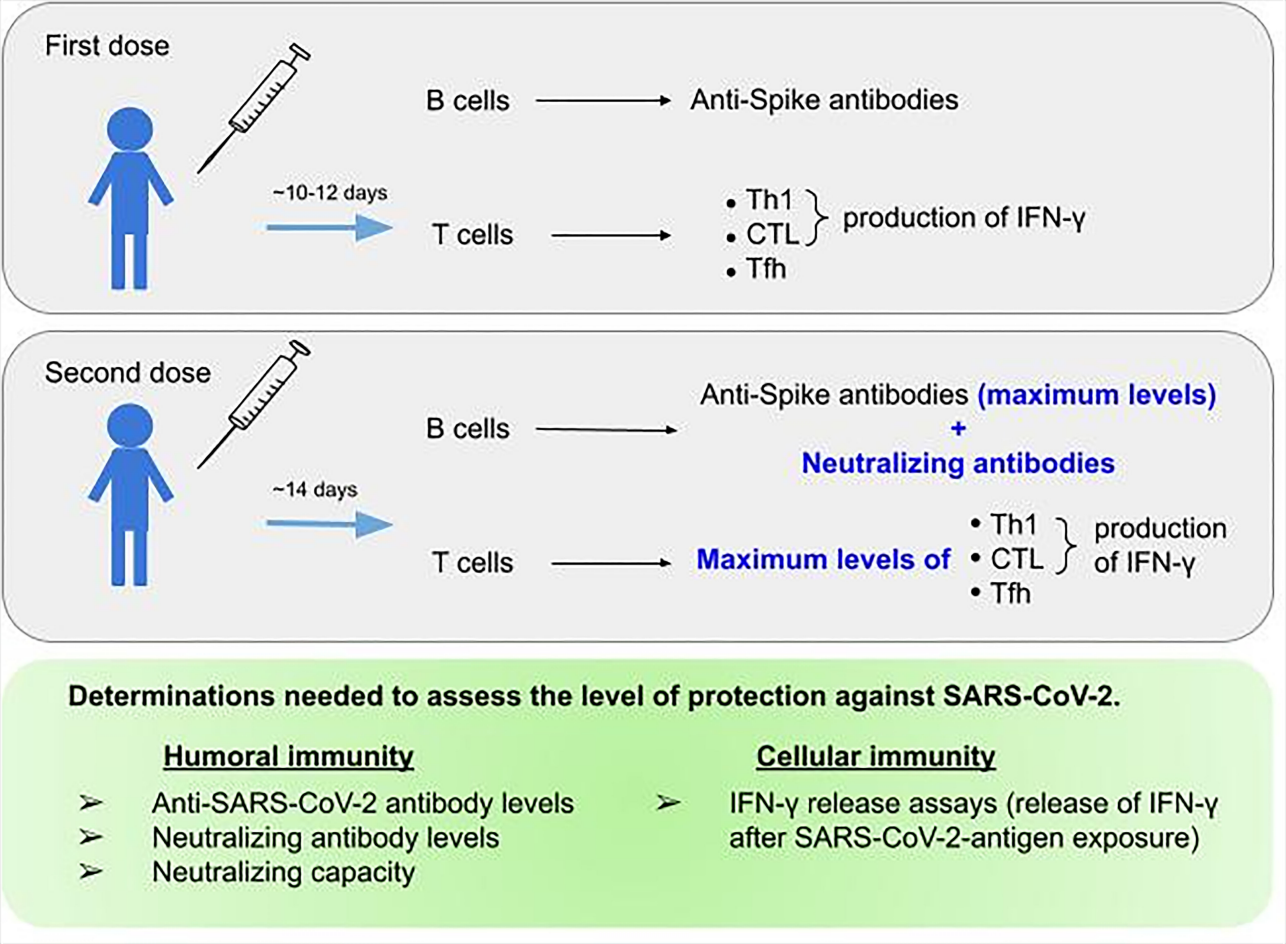
Parameters to be assessed regarding humoral and cellular response to SARS-CoV-2 mRNA vaccine. The top and middle figure outline the immunological response achieved after the first and second doses of mRNA vaccines, respectively. The bottom figure summarizes the parameters needed to assess protection against SARS-CoV-2.

mRNA vaccination leads to the development of both humoral and cellular immunity against the Covid-19 spike protein (249, 250) **Figure 2**. The onset of protection for mRNA vaccines has been observed as early as 10-12 days after the first dose (251) and during this phase T cells and spike-specific antibodies are detectable (250, 252) but neutralizing antibodies do not appear until after the second vaccine dose (253–256). In fact, the presence of anti-S reactive T cells secreting IFN-γ or IL-2 is remarkable as early as three days post-vaccination, but it is not until 14 days after completing the vaccination schedule that they reach their maximum levels (121). The development of humoral responses is gradual and they only consistently reach peak levels after the second vaccination dose (257, 258). In fact, the highest frequencies of spike-binding germinal centre B cells and plasmablasts in draining lymph nodes were reached at twelve weeks after the second immunization (152). However, the natural course of humoral immunity is to decrease over time, with reductions in neutralizing antibody titers (259, 260). At three months post-vaccination, the neutralization capacity was significantly decreased, in agreement with lower S-RBD antibody levels (261) in all variants described to date, from Alpha to Omicron (260). This may, feasibly, be due to the fact that not all vaccine-induced plasmablasts commit or are maintained as long-lived memory plasma cells (123, 262, 263). However, these reductions do not necessarily correspond to proportional reductions in vaccine efficacy over time, and neither do reductions in vaccine efficacy against mild disease necessarily predict reductions in efficacy against severe disease. This may be because protection against severe disease is mediated not only by antibody response, which might be relatively short lived for some vaccines, but also by cell-mediated immunity and memory responses, which are generally longer lived (152). In fact, memory humoral and cellular responses are still detectable in vaccinated individuals who have not undergone COVID-19, and in those who have recovered from COVID-19, eight months after vaccination, despite a progressive decline in antibody levels (230). At six months, although vaccinated individuals show a decreased level of anti-S IgG, all of them present cell-mediated immune responses. The decrease in antibody titers is apparently compensated by an increased neutralization capacity and a robust cellular immune response, which is reflected by a high level of IFN-γ synthesis by the stimulated T-cells (264). In mice, the primary source of serum IFN-γ one day after secondary immunization are CD4+ and CD8+ T cells, which results in improved myeloid cell activation after secondary immunization (211). Given all of the information mentioned above, the assessment of humoral immune response as determined by the measurement of antibodies against the receptor-binding domain of the spike protein after vaccination underestimates the immunogenicity of SARS-CoV-2 vaccines and a combined analysis of humoral and cellular immunity was proposed for the identification of vaccine responders (265).

The type of response that vaccines should trigger must always be Th1 cell response or balanced T-cell responses, because when the response is Th2 cell, it has been associated with enhanced respiratory disease (266–270). Moreover, we have looked at how T cells also play a critical role in B-cell maturation and therefore the induction of a strong and durable antibody response (150). In most of the current COVID-19 vaccines that have reported clinical trial results, the induction of cellular response in humans has a Th1 bias and/or is characterized by IFN-γ expression and appears to be substantially protective against severe disease in all the major viral variants (271). Thus, in a longitudinal study, Painter M. M. et al. (250) show that mRNA vaccines induce Th1 and Tfh cell responses following the first dose, correlated with post-boost CD8+T cells and neutralizing antibodies, respectively, which is expected since Th1 cells predominantly facilitate CD8+ T cell response, while Tfh cells help promote optimal B cell, germinal center, and antibody responses (150, 272–274). At three months, 87% of vaccinated individuals developed either CD4+ or CD8+ T cell responses (261) but after the first dose, in subjects who had no previous contact with SARS-CoV-2, vaccination induced rapid and robust SARS-CoV-2-specific CD4+ T cell responses compared with CD8+T cell responses, which developed gradually and were variable in magnitude (250). However, other authors observed a robust, stable and fully functional response of Spike-specific CD8 T cells after primary vaccination (275). Differences in cohort or methodology may have affected the discrepancy in these findings, but these observations indicate that SARS-CoV-2-specific CD4+ T cells are largely contributors to the protection observed early after the first vaccine dose (251, 276).

CD4+ T cell responses were detected after immunization with ChAdOx-1 S, Ad26.COV2.S, mRNA-1273 or BNT162b2 vaccines, and did not show significant differences between the different variants of concern, including the Omicron variant (143). One or two doses of vaccine elicited a persistent and robust cellular immune memory response even when vaccinated individuals had been infected previously (277–279), with an homogeneity in the magnitude (264). Although previous infected individuals had similar frequencies of vaccine-induced CD4+ T cells as non-infected individuals, the former produced greater IFN-γ following spike-stimulation (230) In addition, T cell reactivity following vaccination or natural infection proved to be similar across early strains (Alpha, Beta and Gamma) given that 93% and 97% of CD4 and CD8 epitopes are 100% conserved across these variants, potentially reducing the severity of COVID-19 if a progression of infection occurs, even though neutralizing antibodies for emerging variants might be reduced in comparison with the original strain of SARS-CoV-2 (280). The same applies to the latest variants, since SARS-CoV-2 vaccination induces immunological CD4+ and CD8+ T cell memory able to cross-recognize variants from Alpha to Omicron (281). Furthermore, CD4+ and CD8+ SARS-CoV-2 spike-specific T cell responses triggered by prior infection with the original strain or BNT 162b2 vaccination, remain largely intact against the Omicron strain (282). This is due to the fact that the vast majority of T cell epitopes are fully conserved (279–281, 283–286), which suggests that the continued evolution of variants has not been associated with increased viral escape from T cell responses at the population level, and HLA binding of the mutated epitopes has been well conserved for the majority of the epitopes in Alpha to Omicron variants (281). Furthermore, it has been proposed that the phenotype of memory and the helper subset distribution of SARS-CoV-2-specific CD4+ and CD8+ T cells responses elicited by second dose vaccine are similar to the ones detected in individuals who have gone through a natural SARS-CoV-2 infection (250).

The effect of the booster dose (a third vaccination dose) is different in naïve individuals than in recovered COVID-19 individuals. In naïve individuals, this booster significantly increased the levels of spike-specific antibodies and B and CD4+ T cells, while in recovered COVID-19 individuals, the booster dose has minor effects (230), in line with what was observed for the second vaccine dose (249, 250). Moreover, the second vaccine dose in individuals who have undergone symptomatic infection with SARS-CoV-2 is associated with lower neutralizing antibody levels, in addition to T and B cell spike-specific frequencies, which suggests that a vaccine over-boost strategy may lead to anergy and exhaustion (249, 257). It is important to differentiate between the immune response driven by the spike protein vaccine and the response resulting from SARS-CoV-2 infection, where the innate immune response triggers the adaptive immune response, and many more elements come into play in a much more complex process. In addition, the effect of repeated doses on overactivation and the role played in its regulatory mechanisms, as well as the appearance of exhausted cells, merits further study.

Age is an important factor influencing vaccine responses, and there have been studies that report elderly people responding poorly to influenza, Hepatitis A and B, and pneumococcal vaccines as they develop lower antibody levels and weaker cell-mediated responses (287). Immunosenescense is likely to affect the vaccine response to SARS-CoV-2, as spike-specific IFN-γ T cell responses to vaccines were impaired in the age group over 80 years (288, 289) and individuals with a higher number of immunosenescent CD8+ T_EMRA_ cells have lower spike-specific CD4+ T cell responses (261). For example, a study of COVID-19-naïve nursing-home residents found that both humoral and cellular responses declined after four weeks and remained lower than those of healthcare workers after 24 weeks (290). These data emphasize the need for additional measures for the fragile elderly population.

Considering this information, to ensure that responses mediated by antibodies with neutralizing capacity are complemented by T cell responses, an alternative parallel strategy in vaccine generation should involve the inclusion of additional antigens and T cell epitopes. This needs to be taken into account since early functional T cells specific to SARS-CoV-2- have a prognostic value with important implications for vaccine design and immune monitoring (125).

## Discussion

SARS-CoV-2, the coronavirus responsible for the last global pandemic, which originated in December 2019, is the causative agent of the disease called COVID-19. The existence of cross-reactivity between the immunity created by the common cold coronaviruses and SARS-CoV-2 has not avoided infection but may have possibly reduced the severity of the disease in some individuals. SARS-CoV-2, like other viruses, has evolved immune evasion mechanisms. In fact, multiple evasion mechanisms have been observed at the level of innate and adaptive humoral immune response; however, the evasion mechanisms involved in cellular response, although existing, require further study.

SARS-CoV-2 infection triggers a potent immune response that directs CD4+ T cellular adaptive response towards Th1 polarization and an activation of CD8+ CTLs, both IFN-γ producers, as well as an antibody-producing humoral response. Different mechanisms cause an imbalance in this response, leading to an overactivation of the innate immune system and resulting in a cytokine storm, together with a hyperactivation of the adaptive immune response that will consequently cause an exhaustion of the latter. The fundamental role played by cellular immunity as the main axis of the immune response against SARS-CoV-2, directing the different components involved, such as the innate response and the humoral response, is evidenced throughout this review.

Many efforts are being made during this pandemic to avoid both primoinfections and reinfections, using massive vaccination strategies. Both natural infection and vaccines produce long-term memory T cells, CD4+ and CD78+, which would protect the population particularly by avoiding severe infections and being associated with a better prognosis. Even so, the immunity provided by vaccination is more limited than the one provided by natural infection. This is because the immune response in vaccination is limited to the spike protein, which affects the variety of the T cell response and, notably, CD8+ T cell memory, which seems to be associated with a better prognosis in the case of non-spike-specific CD8+ T cells. Thus, a future vaccination strategy should include antigens, which are also important for the cellular response, to fill in these gaps.

A successful vaccination strategy requires knowledge of the previous immunity of the individual to SARS-CoV-2 since the cellular immune response to the vaccine is different in naïve individuals and in those who have been previously infected. In the latter, there is a faster and more robust response, which is already detectable with the first vaccine dose. The different studies performed to date make it possible to reach some conclusions concerning the role of cell-mediated immunity in SARS-CoV-2 infection/vaccination (**Table 1**). Hopefully, in the future, the analysis of cellular immunological memory generated by a previous infection or by vaccination will provide us with the tools required to fight against future variants of SARS-CoV-2 in terms of infection control, as well as future revaccination programs.

**Table 1:** Up-to-date key concepts to consider regarding natural infection or vaccination immunity against SARS-CoV-2.

KEY CONCEPTS
The presence of cross-reactivity, either humoral or cellular, between common cold hCoV and SARS-CoV-2 does not prevent infection but may be associated with less severe COVID-19.
The presence of SARS-CoV-2-specific CD4+ Th1 IFN-γ-producing cells and CD8+ CTLs cells were associated with reduced disease severity.
T lymphocyte recruitment to infected lung tissues and T lymphocyte apoptosis/necrosis caused by the cytokine storm might be crucial determinants of CD4+ and CD8+ T-cell lymphopenia in severe COVID-19 cases.
Severe /fatal disease presents with excessive hyperactivation of immune function with increased Tregs and Th2 and/or Th17 cell-biased phenotype, leading to T cell exhaustion and subsequently to a state of anergy.
Functional memory B and T cells to SARS-CoV-2 have been detected 12 months after natural infection. SARS-CoV-2-specific T cell memory may be long lasting given that COVID-19 convalescent patients develop SARS-CoV-2-specific TSCM cells that display a non-exhausted phenotype.
The immunogenicity of SARS-CoV-2 vaccines involves the humoral response (number of spike-specific antibodies, neutralizing antibodies, and antibody neutralization capacity) and the cellular response (IFN-γ-producing CD4+ and CD8+ T cells). Therefore, a combined analysis of humoral and cellular immunity is necessary for the identification of vaccine responders and the immune protection evolution.

## Author Contributions

EM and EL-P contributed to the conception and design of the article, the interpretation of the relevant literature, and the writing of the manuscript and prepared the figures. PD contributed to the critical revision of the article for intellectual content and funding acquisition. All authors contributed to the article and approved the submitted version.

## Funding

This work was partially supported by the grant COV20/00070 (to PD), Instituto de Salud Carlos III, Madrid, Spain. PD is supported by a grant from the Programa de Intensificación de Investigadores (INT19/00036)-ISCIII.

## Conflict of Interest

The authors declare that the research was conducted in the absence of any commercial or financial relationships that could be construed as a potential conflict of interest.

## Publisher’s Note

All claims expressed in this article are solely those of the authors and do not necessarily represent those of their affiliated organizations, or those of the publisher, the editors and the reviewers. Any product that may be evaluated in this article, or claim that may be made by its manufacturer, is not guaranteed or endorsed by the publisher.

## References

[1] Jakubowiak AJ Dytfeld D Griffith KA Lebovic D Vesole DH Jagannath S. A Phase 1/2 Study of Carfilzomib in Combination With Lenalidomide and Low-Dose Dexamethasone as a Frontline Treatment for Multiple Myeloma. Blood (2012) 120(9):1801–9. doi: 10.1182/blood-2012-04-422683 PMC5162553 22665938

[2] Masters PS. The Molecular Biology of Coronaviruses. Adv Virus Res (2006) 65:193–292. doi: 10.1016/S0065-3527(06)66005-3 PMC7112330 16877062

[3] Zhong NS Zheng BJ Li YM Poon LLM Xie ZH Chan KH. Epidemiology and Cause of Severe Acute Respiratory Syndrome (SARS) in Guangdong, People’s Republic of China, in February, 2003. Lancet (2003) 362(9393):1353–8. doi: 10.1016/S0140-6736(03)14630-2 PMC7112415 14585636

[4] Lee N Hui D Wu A Chan P Cameron P Joynt GM. A Major Outbreak of Severe Acute Respiratory Syndrome in Hong Kong. N Engl J Med (2003) 348(20):1986–94. doi: 10.1056/NEJMoa030685 12682352

[5] Zaki AM van Boheemen S Bestebroer TM Osterhaus ADME Fouchier RAM. Isolation of a Novel Coronavirus From a Man With Pneumonia in Saudi Arabia. N Engl J Med (2012) 367(19):1814–20. doi: 10.1056/NEJMoa1211721 23075143

[6] de Groot RJ Baker SC Baric RS Brown CS Drosten C Enjuanes L. Middle East Respiratory Syndrome Coronavirus (MERS-CoV): Announcement of the Coronavirus Study Group. J Virol (2013) 87(14):7790–2. doi: 10.1128/JVI.01244-13 PMC3700179 23678167

[7] Zhu N Zhang D Wang W Li X Yang B Song J. A Novel Coronavirus From Patients With Pneumonia in China, 2019. N Engl J Med (2020) 382(8):727–33. doi: 10.1056/NEJMoa2001017 PMC7092803 31978945

[8] Siddell SG Walker PJ Lefkowitz EJ Mushegian AR Adams MJ Dutilh BE. Additional Changes to Taxonomy Ratified in a Special Vote by the International Committee on Taxonomy of Viruses (October 2018). Arch Virol (2019) 164(3):943–6. doi: 10.1007/s00705-018-04136-2 30663020

[9] Gorbalenya AE Baker SC Baric RS de Groot RJ Drosten C Gulyaeva AA. The Species Severe Acute Respiratory Syndrome-Related Coronavirus: Classifying 2019-Ncov and Naming it SARS-CoV-2. Nat Microbiol (2020) 5(4):536–44. doi: 10.1038/s41564-020-0695-z PMC7095448 32123347

[10] Woo PCY Lau SKP Lam CSF Lau CCY Tsang AKL Lau JHN. Discovery of Seven Novel Mammalian and Avian Coronaviruses in the Genus Deltacoronavirus Supports Bat Coronaviruses as the Gene Source of Alphacoronavirus and Betacoronavirus and Avian Coronaviruses as the Gene Source of Gammacoronavirus and Deltacoronavi. J Virol (2012) 86(7):3995–4008. doi: 10.1128/JVI.06540-11 22278237 PMC3302495

[11] Lu R Zhao X Li J Niu P Yang B Wu H. Genomic Characterisation and Epidemiology of 2019 Novel Coronavirus: Implications for Virus Origins and Receptor Binding. Lancet (2020) 395(10224):565–74. doi: 10.1016/S0140-6736(20)30251-8 PMC7159086 32007145

[12] Zhou P Yang XL Wang XG Hu B Zhang L Zhang W. A Pneumonia Outbreak Associated With a New Coronavirus of Probable Bat Origin. Nature (2020) 579(7798):270–3. doi: 10.1038/s41586-020-2012-7 PMC7095418 32015507

[13] Callaway E. Beyond Omicron: What’s Next for COVID’s Viral Evolution. Nature (2021) 600(7888):204–7. doi: 10.1038/d41586-021-03619-8 34876665

[14] Harvey WT Carabelli AM Jackson B Gupta RK Thomson EC Harrison EM. SARS-CoV-2 Variants, Spike Mutations and Immune Escape. Nat Rev Microbiol (2021) 19(7):409–24. doi: 10.1038/s41579-021-00573-0 PMC8167834 34075212

[15] Walensky RP Walke HT Fauci AS. SARS-CoV-2 Variants of Concern in the United States-Challenges and Opportunities. JAMA - J Am Med Assoc (2021) 325(11):1037–8. doi: 10.1001/jama.2021.2294 PMC9009864 33595644

[16] Uriu K Kimura I Shirakawa K Takaori-Kondo A Nakada T Kaneda A. Neutralization of the SARS-CoV-2 Mu Variant by Convalescent and Vaccine Serum. N Engl J Med (2021) 385(25):2397–9. doi: 10.1056/NEJMc2114706 PMC8609602 34731554

[17] Mlcochova P Kemp S Dhar MS Papa G Meng B Ferreira IATM. SARS-CoV-2 B.1.617.2 Delta Variant Replication and Immune Evasion. Nature (2021) 599:114–9. doi: 10.1038/s41586-021-03944-y PMC8566220 34488225

[18] Karim SSA Karim QA. Omicron SARS-CoV-2 Variant: A New Chapter in the COVID-19 Pandemic. Lancet (2021) 398(10317):2126–8. doi: 10.1016/S0140-6736(21)02758-6 PMC8640673 34871545

[19] St. John AL Rathore APS. Adaptive Immune Responses to Primary and Secondary Dengue Virus Infections. Nat Rev Immunol (2019) 19(4):218–30. doi: 10.1038/s41577-019-0123-x 30679808

[20] Hayward AC Wang L Goonetilleke N Fragaszy EB Bermingham A Copas A. Natural T Cell-Mediated Protection Against Seasonal and Pandemic Influenza: Results of the Flu Watch Cohort Study. Am J Respir Crit Care Med (2015) 191(12):1422–31. doi: 10.1164/rccm.201411-1988OC PMC4476562 25844934

[21] Greenbaum JA Kotturi MF Kim Y Oseroff C Vaughan K Salimi N. Pre-Existing Immunity Against Swine-Origin H1N1 Influenza Viruses in the General Human Population. Proc Natl Acad Sci U S A (2009) 106(48):20365–70. doi: 10.1073/pnas.0911580106 PMC2777968 19918065

[22] Sridhar S Begom S Bermingham A Hoschler K Adamson W Carman W. Cellular Immune Correlates of Protection Against Symptomatic Pandemic Influenza. Nat Med (2013) 19(10):1305–12. doi: 10.1038/nm.3350 24056771

[23] Wilkinson TM Li CKF Chui CSC Huang AKY Perkins M Liebner JC. Preexisting Influenza-Specific CD4 + T Cells Correlate With Disease Protection Against Influenza Challenge in Humans. Nat Med (2012) 18(2):274–80. doi: 10.1038/nm.2612 22286307

[24] Weber F Wagner V Rasmussen SB Hartmann R Paludan SR. Double-Stranded RNA Is Produced by Positive-Strand RNA Viruses and DNA Viruses But Not in Detectable Amounts by Negative-Strand RNA Viruses. J Virol (2006) 80(10):5059–64. doi: 10.1128/JVI.80.10.5059-5064.2006 PMC1472073 16641297

[25] Zielecki F Weber M Eickmann M Spiegelberg L Zaki AM Matrosovich M. Human Cell Tropism and Innate Immune System Interactions of Human Respiratory Coronavirus EMC Compared to Those of Severe Acute Respiratory Syndrome Coronavirus. J Virol (2013) 87(9):5300–4. doi: 10.1128/JVI.03496-12 PMC3624328 23449793

[26] Rasmussen SB Reinert LS Paludan SR. Innate Recognition of Intracellular Pathogens: Detection and Activation of the First Line of Defense. Apmis (2009) 117(5–6):323–37. doi: 10.1111/j.1600-0463.2009.02456.x 19400860

[27] Yim HCH Williams BRG. Protein Kinase R and the Inflammasome. J Interf Cytokine Res (2014) 34(6):447–54. doi: 10.1089/jir.2014.0008 24905201

[28] Yoneyama M Jogi M Onomoto K. Regulation of Antiviral Innate Immune Signaling by Stress-Induced RNA Granules. J Biochem (2015) 159(3):279–86. doi: 10.1093/jb/mvv122 PMC4763080 26748340

[29] Lund JM Alexopoulou L Sato A Karow M Adams NC Gale NW. Recognition of Single-Stranded RNA Viruses by Toll-Like Receptor 7. Proc Natl Acad Sci U S A (2004) 101(15):5598–603. doi: 10.1073/pnas.0400937101 PMC397437 15034168

[30] De Wit E Van Doremalen N Falzarano D Munster VJ. SARS and MERS: Recent Insights Into Emerging Coronaviruses. Nat Rev Microbiol (2016) 14(8):523–34. doi: 10.1038/nrmicro.2016.81 PMC7097822 27344959

[31] de Wilde AH Snijder EJ Kikkert M van Hemert MJ. Host Factors in Coronavirus Replication. Curr Top Microbiol Immunol (2018) 419:1–42. doi: 10.1007/82_2017_25 28643204 PMC7119980

[32] Ratia K Kilianski A Baez-Santos YM Baker SC Mesecar A. Structural Basis for the Ubiquitin-Linkage Specificity and Deisgylating Activity of SARS-CoV Papain-Like Protease. PLoS Pathog (2014) 10(5):e1004113. doi: 10.1371/journal.ppat.1004113 24854014 PMC4031219

[33] Devaraj SG Wang N Chen Z Chen Z Tseng M Barretto N. Regulation of IRF-3-Dependent Innate Immunity by the Papain-Like Protease Domain of the Severe Acute Respiratory Syndrome Coronavirus. J Biol Chem (2007) 282(44):32208–21. doi: 10.1074/jbc.M704870200 PMC2756044 17761676

[34] Frieman M Ratia K Johnston RE Mesecar AD Baric RS. Severe Acute Respiratory Syndrome Coronavirus Papain-Like Protease Ubiquitin-Like Domain and Catalytic Domain Regulate Antagonism of IRF3 and NF-κb Signaling. J Virol (2009) 83(13):6689–705. doi: 10.1128/JVI.02220-08 PMC2698564 19369340

[35] Sun L Xing Y Chen X Zheng Y Yang Y Nichols DB. Coronavirus Papain-Like Proteases Negatively Regulate Antiviral Innate Immune Response Through Disruption of STING-Mediated Signaling. PLoS One (2012) 7(2):e30802. doi: 10.1371/journal.pone.0030802 22312431 PMC3270028

[36] de Wilde AH Raj VS Oudshoorn D Bestebroer TM van Nieuwkoop S Limpens RWAL. MERS-Coronavirus Replication Induces Severe In Vitro Cytopathology and Is Strongly Inhibited by Cyclosporin A or Interferon-α Treatment. J Gen Virol (2013) 94(PART8):1749–60. doi: 10.1099/vir.0.052910-0 PMC3749523 23620378

[37] Knoops K Kikkert M Van Den Worm SHE Zevenhoven-Dobbe JC van der Meer Y Koster AJ. SARS-Coronavirus Replication Is Supported by a Reticulovesicular Network of Modified Endoplasmic Reticulum. PLoS Biol (2008) 6(9):1957–74. doi: 10.1371/journal.pbio.0060226 PMC2535663 18798692

[38] Siu KL Chan CP Kok KH Chiu-Yat Woo P Jin DY. Suppression of Innate Antiviral Response by Severe Acute Respiratory Syndrome Coronavirus M Protein is Mediated Through the First Transmembrane Domain. Cell Mol Immunol (2014) 11(2):141–9. doi: 10.1038/cmi.2013.61 PMC4003381 24509444

[39] Lui PY Wong LYR Fung CL Siu KL Yeung ML Yuen KS. Middle East Respiratory Syndrome Coronavirus M Protein Suppresses Type I Interferon Expression Through the Inhibition of TBK1-Dependent Phosphorylation of IRF3. Emerg Microbes Infect (2016) 5(1):1–9. doi: 10.1038/emi.2016.33 PMC4855074 27094905

[40] Siu KL Kok KH Ng MHJ Poon VKM Yuen KY Zheng BJ. Severe Acute Respiratory Syndrome Coronavirus M Protein Inhibits Type I Interferon Production by Impeding Theformation of TRAF3·TANK·Tbk1/Ikkϵ Complex. J Biol Chem (2009) 284(24):16202–9. doi: 10.1074/jbc.M109.008227 PMC2713514 19380580

[41] Drosten C Meyer B Müller MA Corman VM Al-Masri M Hossain R. Transmission of MERS-Coronavirus in Household Contacts. N Engl J Med (2014) 371(9):828–35. doi: 10.1056/NEJMoa1405858 25162889

[42] Zhao J Alshukairi AN Baharoon SA Ahmed WA Bokhari AA Nehdi AM. Recovery From the Middle East Respiratory Syndrome is Associated With Antibody and T Cell Responses. Sci Immunol (2017) 2(14):eaan5393. doi: 10.1126/sciimmunol.aan5393 28778905 PMC5576145

[43] Chu H Zhou J Wong BHY Li C Chan JFW Cheng ZS. Middle East Respiratory Syndrome Coronavirus Efficiently Infects Human Primary T Lymphocytes and Activates the Extrinsic and Intrinsic Apoptosis Pathways. J Infect Dis (2016) 213(6):904–14. doi: 10.1093/infdis/jiv380 PMC7107330 26203058

[44] Yang Y Xiong Z Zhang S Yan Y Nguyen J Ng B. Bcl-xL Inhibits T-Cell Apoptosis Induced by Expression of SARS Coronavirus E Protein in the Absence of Growth Factors. Biochem J (2005) 392(1):135–43. doi: 10.1042/BJ20050698 PMC1317672 16048439

[45] Cameron MJ Bermejo-Martin JF Danesh A Muller MP Kelvin DJ. Human Immunopathogenesis of Severe Acute Respiratory Syndrome (SARS). Virus Res (2008) 133(1):13–9. doi: 10.1016/j.virusres.2007.02.014 PMC7114310 17374415

[46] He Z Zhao C Dong Q Zhuang H Song S Peng G. Effects of Severe Acute Respiratory Syndrome (SARS) Coronavirus Infection on Peripheral Blood Lymphocytes and Their Subsets. Int J Infect Dis (2005) 9(6):323–30. doi: 10.1016/j.ijid.2004.07.014 PMC7110876 16095942

[47] Li CK Wu H Yan H Ma S Wang L Zhang M. T Cell Responses to Whole SARS Coronavirus in Humans. J Immunol (2008) 181(8):5490–500. doi: 10.4049/jimmunol.181.8.5490 PMC2683413 18832706

[48] Fan YY Huang ZT Li L Wu MH Yu T Koup RA. Characterization of SARS-CoV-Specific Memory T Cells From Recovered Individuals 4 Years After Infection. Arch Virol (2009) 154(7):1093–9. doi: 10.1007/s00705-009-0409-6 PMC2796960 19526193

[49] Oh H-LJ Chia A Chang CXL Leong HN Ling KL Grotenbreg GM. Engineering T Cells Specific for a Dominant Severe Acute Respiratory Syndrome Coronavirus CD8 T Cell Epitope. J Virol (2011) 85(20):10464–71. doi: 10.1128/JVI.05039-11 PMC3187484 21813600

[50] Tang F Quan Y Xin Z-T Wrammert J Ma M-J Lv H. Lack of Peripheral Memory B Cell Responses in Recovered Patients With Severe Acute Respiratory Syndrome: A Six-Year Follow-Up Study. J Immunol (2011) 186(12):7264–8. doi: 10.4049/jimmunol.0903490 21576510

[51] Ng OW Chia A Tan AT Jadi RS Leong HN Bertoletti A. Memory T Cell Responses Targeting the SARS Coronavirus Persist Up to 11 Years Post-Infection. Vaccine (2016) 34(17):2008–14. doi: 10.1016/j.vaccine.2016.02.063 PMC7115611 26954467

[52] Gorse GJ Patel GB Vitale JN O’Connor TZ. Prevalence of Antibodies to Four Human Coronaviruses is Lower in Nasal Secretions Than in Serum. Clin Vaccine Immunol (2010) 17(12):1875–80. doi: 10.1128/CVI.00278-10 PMC3008199 20943876

[53] Grifoni A Weiskopf D Ramirez SI Mateus J Dan JM Moderbacher CR. Targets of T Cell Responses to SARS-CoV-2 Coronavirus in Humans With COVID-19 Disease and Unexposed Individuals. Cell (2020) 181(7):1489–501.e15. doi: 10.1016/j.cell.2020.05.015 32473127 PMC7237901

[54] Weiskopf D Schmitz KS Raadsen MP Grifoni A Okba NMA Endeman H. Phenotype and Kinetics of SARS-CoV-2-Specific T Cells in COVID-19 Patients With Acute Respiratory Distress Syndrome. Sci Immunol (2020) 5(48):eabd2071. doi: 10.1126/sciimmunol.abd2071 32591408 PMC7319493

[55] Braun J Loyal L Frentsch M Wendisch D Georg P Kurth F. SARS-CoV-2-Reactive T Cells in Healthy Donors and Patients With COVID-19. Nature (2020) 587(7833):270–4. doi: 10.1038/s41586-020-2598-9 32726801

[56] Le Bert N Tan AT Kunasegaran K Tham CYL Hafezi M Chia A. SARS-CoV-2-Specific T Cell Immunity in Cases of COVID-19 and SARS, and Uninfected Controls. Nature (2020) 584(7821):457–62. doi: 10.1038/s41586-020-2550-z 32668444

[57] Meckiff BJ Ramírez-Suástegui C Fajardo V Chee SJ Kusnadi A Simon H. Imbalance of Regulatory and Cytotoxic SARS-CoV-2-Reactive CD4+ T Cells in COVID-19. Cell (2020) 183(5):1340–53.e16. doi: 10.1016/j.cell.2020.10.001 33096020 PMC7534589

[58] Long QX Liu BZ Deng HJ Wu GC Deng K Chen YK. Antibody Responses to SARS-CoV-2 in Patients With COVID-19. Nat Med (2020) 26(6):845–8. doi: 10.1038/s41591-020-0897-1 32350462

[59] Nguyen-Contant P Embong AK Kanagaiah P Chaves FA Yang H Branche AR. S Protein-Reactive IGG and Memory B Cell Production After Human SARS-CoV-2 Infection Includes Broad Reactivity to the S2 Subunit. MBio (2020) 11(5):1–11. doi: 10.1128/mBio.01991-20 PMC7520599 32978311

[60] Ng KW Faulkner N Cornish GH Rosa A Harvey R Hussain S. Preexisting and *De Novo* Humoral Immunity to SARS-CoV-2 in Humans. Sci (80- ) (2020) 370(6522):1339–43. doi: 10.1126/science.abe1107 PMC7857411 33159009

[61] Anderson EM Goodwin EC Verma A Arevalo CP Bolton MJ Weirick ME. Seasonal Human Coronavirus Antibodies are Boosted Upon SARS-CoV-2 Infection But Not Associated With Protection. Cell (2021) 184(7):1858–64.e10. doi: 10.1016/j.cell.2021.02.010 33631096 PMC7871851

[62] Poston D Weisblum Y Wise H Templeton K Jenks S Hatziioannou T. Absence of Severe Acute Respiratory Syndrome Coronavirus 2 Neutralizing Activity in Prepandemic Sera From Individuals With Recent Seasonal Coronavirus Infection. Clin Infect Dis (2021) 73(5):E1208–11. doi: 10.1093/cid/ciaa1803 PMC7799301 33270134

[63] Shrwani K Sharma R Krishnan M Jones T Mayora-Neto M Cantoni D. Detection of Serum Cross-Reactive Antibodies and Memory Response to SARS-CoV-2 in Prepandemic and Post-COVID-19 Convalescent Samples. J Infect Dis (2021) 224(8):1305–15. doi: 10.1093/infdis/jiab333 PMC8557674 34161567

[64] Mateus J Grifoni A Tarke A Sidney J Ramirez SI Dan JM. Selective and Cross-Reactive SARS-CoV-2 T Cell Epitopes in Unexposed Humans. Sci (2020) 370(6512):89–94. doi: 10.1126/science.abd3871 PMC7574914 32753554

[65] Sette A Crotty S. Pre-Existing Immunity to SARS-CoV-2: The Knowns and Unknowns. Nat Rev Immunol (2020) 20(8):457–8. doi: 10.1038/s41577-020-0389-z PMC7339790 32636479

[66] Saletti G Gerlach T Jansen JM Molle A Elbahesh H Ludlow M. Older Adults Lack SARS CoV-2 Cross-Reactive T Lymphocytes Directed to Human Coronaviruses OC43 and NL63. Sci Rep (2020) 10(1):21447. doi: 10.1038/s41598-020-78506-9 33293664 PMC7722724

[67] Nelde A Bilich T Heitmann JS Maringer Y Salih HR Roerden M. SARS-CoV-2-Derived Peptides Define Heterologous and COVID-19-Induced T Cell Recognition. Nat Immunol (2021) 22(1):74–85. doi: 10.1038/s41590-020-00808-x 32999467

[68] Schulien I Kemming J Oberhardt V Wild K Seidel LM Killmer S. Characterization of Pre-Existing and Induced SARS-CoV-2-Specific CD8+ T Cells. Nat Med (2021) 27(1):78–85. doi: 10.1038/s41591-020-01143-2 33184509

[69] Bacher P Rosati E Esser D Martini GR Saggau C Schiminsky E. Low-Avidity CD4+ T Cell Responses to SARS-CoV-2 in Unexposed Individuals and Humans With Severe COVID-19. Immunity (2020) 53(6):1258–71.e5. doi: 10.1016/j.immuni.2020.11.016 33296686 PMC7689350

[70] Sagar M Reifler K Rossi M Miller NS Sinha P White LF. Recent Endemic Coronavirus Infection is Associated With Less-Severe COVID-19. J Clin Invest (2021) 131(1):e143380. doi: 10.1172/JCI143380 PMC7773342 32997649

[71] Loyal L Braun J Henze L Kruse B Dingeldey M Reimer U. Cross-Reactive CD4+ T Cells Enhance SARS-CoV-2 Immune Responses Upon Infection and Vaccination. Sci (80- ) (2021) 374(6564):eabh1823. doi: 10.1126/science.abh1823 PMC10026850 34465633

[72] Viswanathan T Arya S Chan SH Qi S Dai N Misra A. Structural Basis of RNA Cap Modification by SARS-CoV-2. Nat Commun (2020) 11(1):3718. doi: 10.1038/s41467-020-17496-8 32709886 PMC7381649

[73] Rosas-Lemus M Minasov G Shuvalova L Inniss NL Kiryukhina O Brunzelle J. High-Resolution Structures of the SARS-CoV-2 2′-O-Methyltransferase Reveal Strategies for Structure-Based Inhibitor Design. Sci Signal (2020) 13(651):eabe1202. doi: 10.1126/scisignal.abe1202 32994211 PMC8028745

[74] Gordon DE Jang GM Bouhaddou M Xu J Obernier K White KM. A SARS-CoV-2 Protein Interaction Map Reveals Targets for Drug Repurposing. Nature (2020) 583(7816):459–68. doi: 10.1038/s41586-020-2286-9 PMC7431030 32353859

[75] Wang C Chen T Zhang J Yang M Li N Xu X. The E3 Ubiquitin Ligase Nrdp1 “Preferentially” Promotes TLR-Mediated Production of Type I Interferon. Nat Immunol (2009) 10(7):744–52. doi: 10.1038/ni.1742 19483718

[76] Gordon DE Hiatt J Bouhaddou M Rezelj VV Ulferts S Braberg H. Comparative Host-Coronavirus Protein Interaction Networks Reveal Pan-Viral Disease Mechanisms. Science (2020) 370(6521):eabe9403. doi: 10.1126/science.abe9403 33060197 PMC7808408

[77] Vazquez C Swanson SE Negatu SG Dittmar M Miller J Ramage HR. SARS-CoV-2 Viral Proteins NSP1 and NSP13 Inhibit Interferon Activation Through Distinct Mechanisms. PLoS One (2021) 16(6):e0253089. doi: 10.1371/journal.pone.0253089 34166398 PMC8224853

[78] Han L Zhuang MW Deng J Zheng Y Zhang J Nan ML. SARS-CoV-2 ORF9b Antagonizes Type I and III Interferons by Targeting Multiple Components of the RIG-I/MDA-5–MAVS, TLR3–TRIF, and cGAS–STING Signaling Pathways. J Med Virol (2021) 93(9):5376–89. doi: 10.1002/jmv.27050 PMC8242602 33913550

[79] Lei X Dong X Ma R Wang W Xiao X Tian Z. Activation and Evasion of Type I Interferon Responses by SARS-CoV-2. Nat Commun (2020) 11(1):3810. doi: 10.1038/s41467-020-17665-9 32733001 PMC7392898

[80] Wang W Zhou Z Xiao X Tian Z Dong X Wang C. SARS-CoV-2 Nsp12 Attenuates Type I Interferon Production by Inhibiting IRF3 Nuclear Translocation. Cell Mol Immunol (2021) 18(4):945–53. doi: 10.1038/s41423-020-00619-y PMC7907794 33637958

[81] Gao X Zhu K Qin B Olieric V Wang M Cui S. Crystal Structure of SARS-CoV-2 Orf9b in Complex With Human TOM70 Suggests Unusual Virus-Host Interactions. Nat Commun (2021) 12(1):2843. doi: 10.1038/s41467-021-23118-8 33990585 PMC8121815

[82] Jiang HW Zhang HN Meng QF Xie J Li Y Chen H. SARS-CoV-2 Orf9b Suppresses Type I Interferon Responses by Targeting TOM70. Cell Mol Immunol (2020) 17(9):998–1000. doi: 10.1038/s41423-020-0514-8 32728199 PMC7387808

[83] Wu J Shi Y Pan X Wu S Hou R Zhang Y. SARS-CoV-2 ORF9b Inhibits RIG-I-MAVS Antiviral Signaling by Interrupting K63-Linked Ubiquitination of NEMO. Cell Rep (2021) 34(7):108761. doi: 10.1016/j.celrep.2021.108761 33567255 PMC7857071

[84] Liu GQ Lee JH Parker ZM Acharya D Chiang JJ van Gent M. ISG15-Dependent Activation of the Sensor MDA5 is Antagonized by the SARS-CoV-2 Papain-Like Protease to Evade Host Innate Immunity. Nat Microbiol (2021) 6(4):467–78. doi: 10.1038/s41564-021-00884-1 PMC8103894 33727702

[85] Moustaqil M Ollivier E Chiu HP Van Tol S Rudolffi-Soto P Stevens C. SARS-CoV-2 Proteases PLpro and 3clpro Cleave IRF3 and Critical Modulators of Inflammatory Pathways (NLRP12 and TAB1): Implications for Disease Presentation Across Species. Emerg Microbes Infect (2021) 10(1):178–95. doi: 10.1080/22221751.2020.1870414 PMC7850364 33372854

[86] Zheng Y Zhuang MW Han L Zhang J Nan ML Zhan P. Severe Acute Respiratory Syndrome Coronavirus 2 (SARS-CoV-2) Membrane (M) Protein Inhibits Type I and III Interferon Production by Targeting RIG-I/MDA-5 Signaling. Signal Transduct Target Ther (2020) 5(1):299. doi: 10.1038/s41392-020-00438-7 33372174 PMC7768267

[87] Fu YZ Wang SY Zheng ZQ Huang Y Li WW Xu ZS. SARS-CoV-2 Membrane Glycoprotein M Antagonizes the MAVS-Mediated Innate Antiviral Response. Cell Mol Immunol (2021) 18(3):613–20. doi: 10.1038/s41423-020-00571-x PMC7588591 33110251

[88] Xia H Cao Z Xie X Zhang X Chen JYC Wang H. Evasion of Type I Interferon by SARS-CoV-2. Cell Rep (2020) 33(1):108234. doi: 10.1016/j.celrep.2020.108234 32979938 PMC7501843

[89] Hayn M Hirschenberger M Koepke L Nchioua R Straub JH Klute S. Systematic Functional Analysis of SARS-CoV-2 Proteins Uncovers Viral Innate Immune Antagonists and Remaining Vulnerabilities. Cell Rep (2021) 35(7):109126. doi: 10.1016/j.celrep.2021.109126 33974846 PMC8078906

[90] Collier DA De Marco A Ferreira IATM Meng B Datir R Walls AC. SARS-CoV-2 B.1.1.7 Sensitivity to mRNA Vaccine-Elicited, Convalescent and Monoclonal Antibodies. medRxiv Prepr Serv Heal Sci (2021) 593:136–41. doi: 10.1038/s41586-021-03412-7 PMC7616976 33706364

[91] Graham C Seow J Huettner I Khan H Kouphou N Acors S. Neutralization Potency of Monoclonal Antibodies Recognizing Dominant and Subdominant Epitopes on SARS-CoV-2 Spike is Impacted by the B.1.1.7 Variant. Immunity (2021) 54(6):1276–89.e6. doi: 10.1016/j.immuni.2021.03.023 33836142 PMC8015430

[92] Wibmer CK Ayres F Hermanus T Madzivhandila M Kgagudi P Oosthuysen B. SARS-CoV-2 501y.V2 Escapes Neutralization by South African COVID-19 Donor Plasma. Nat Med (2021) 27(4):622–5. doi: 10.1038/s41591-021-01285-x 33654292

[93] Li Q Nie J Wu J Zhang L Ding R Wang H. SARS-CoV-2 501y.V2 Variants Lack Higher Infectivity But do Have Immune Escape. Cell (2021) 184(9):2362–71.e9. doi: 10.1016/j.cell.2021.02.042 33735608 PMC7901273

[94] Wang P Casner RG Nair MS Wang M Yu J Cerutti G. Increased Resistance of SARS-CoV-2 Variant P.1 to Antibody Neutralization. Cell Host Microbe (2021) 29(5):747–51.e4. doi: 10.1016/j.chom.2021.04.007 33887205 PMC8053237

[95] Hoffmann M Arora P Groß R Seidel A Hörnich BF Hahn AS. SARS-CoV-2 Variants B.1.351 and P.1 Escape From Neutralizing Antibodies. Cell (2021) 184(9):2384–93.e12. doi: 10.1016/j.cell.2021.03.036 33794143 PMC7980144

[96] Garcia-Beltran WF Lam EC St. Denis K Nitido AD Garcia ZH Hauser BM. Multiple SARS-CoV-2 Variants Escape Neutralization by Vaccine-Induced Humoral Immunity. Cell (2021) 184(9):2372–83.e9. doi: 10.1016/j.cell.2021.03.013 33743213 PMC7953441

[97] Deng X Garcia-Knight MA Khalid MM Servellita V Wang C Morris MK. Transmission, Infectivity, and Antibody Neutralization of an Emerging SARS-CoV-2 Variant in California Carrying a L452R Spike Protein Mutation. medRxiv (2021) 9:2021.03.07.21252647. doi: 10.1101/2021.03.07.21252647

[98] Planas D Veyer D Baidaliuk A Staropoli I Guivel-Benhassine F Rajah MM. Reduced Sensitivity of SARS-CoV-2 Variant Delta to Antibody Neutralization. Nature (2021) 596(7871):276–80. doi: 10.1038/s41586-021-03777-9 34237773

[99] Schmidt F Muecksch F Weisblum Y Da Silva J Bednarski E Cho A. Plasma Neutralization of the SARS-CoV-2 Omicron Variant. N Engl J Med (2022) 386(6):599–601. doi: 10.1056/NEJMc2119641 35030645 PMC8757565

[100] Zou J Xia H Xie X Kurhade C Machado RRG Weaver SC. Neutralization Against Omicron SARS-CoV-2 From Previous non-Omicron Infection. Cell Host Microbe (2022) 30(4):485–8.e3. doi: 10.1038/s41467-022-28544-w PMC8853806 35245438

[101] Planas D Saunders N Maes P Guivel-Benhassine F Planchais C Buchrieser J. Considerable Escape of SARS-CoV-2 Omicron to Antibody Neutralization. Nature (2021) 602: 671–67. doi: 10.1038/s41586-021-04389-z 35016199

[102] Wang Y Zhang L Li Q Liang Z Li T Liu S. The Significant Immune Escape of Pseudotyped SARS-CoV-2 Variant Omicron. Emerg Microbes Infect (2022) 11(1):1–5. doi: 10.1080/22221751.2021.2017757 34890524 PMC8725892

[103] Fenrich M Mrdenovic S Balog M Tomic S Zjalic M Roncevic A. SARS-CoV-2 Dissemination Through Peripheral Nerves Explains Multiple Organ Injury. Front Cell Neurosci (2020) 14. doi: 10.3389/fncel.2020.00229 PMC7419602 32848621

[104] Tarke A Sidney J Methot N Zhang Y Dan JM Goodwin B. Negligible Impact of SARS-CoV-2 Variants on CD4 + and CD8 + T Cell Reactivity in COVID-19 Exposed Donors and Vaccinees. bioRxiv (2021) 1:2021.02.27.433180. doi: 10.1101/2021.02.27.433180

[105] Tarke A Sidney J Kidd CK Dan JM Ramirez SI Yu ED. Comprehensive Analysis of T Cell Immunodominance and Immunoprevalence of SARS-CoV-2 Epitopes in COVID-19 Cases. Cell Rep Med (2021) 2(2):100204. doi: 10.1016/j.xcrm.2021.100204 33521695 PMC7837622

[106] Ferretti AP Kula T Wang Y Nguyen DMV Weinheimer A Dunlap GS. Unbiased Screens Show CD8+ T Cells of COVID-19 Patients Recognize Shared Epitopes in SARS-CoV-2 That Largely Reside Outside the Spike Protein. Immunity (2020) 53(5):1095–107.e3. doi: 10.1016/j.immuni.2020.10.006 33128877 PMC7574860

[107] Gallagher KME Leick MB Larson RC Berger TR Katsis K Yam JY. MGH COVID-19 Collection & Processing Team, Maus MV. SARS -CoV-2 T-Cell Immunity to Variants of Concern Following Vaccination. bioRxiv [Preprint] (2021) 3:2021.05.03.442455. doi: 10.1101/2021.05.03.442455

[108] Zhang H Deng S Ren L Zheng P Hu X Jin T. Profiling CD8+ T Cell Epitopes of COVID-19 Convalescents Reveals Reduced Cellular Immune Responses to SARS-CoV-2 Variants. Cell Rep (2021) 36(11):109708. doi: 10.1016/j.celrep.2021.109708 34506741 PMC8390359

[109] Codo AC Davanzo GG de Brito Monteiro L de Souza GF Muraro SP Virgilio-da-Silva JV. Elevated Glucose Levels Favor SARS-CoV-2 Infection and Monocyte Response Through a HIF-1α/Glycolysis-Dependent Axis. Cell Metab (2020) 32(3):437–46.e5. doi: 10.2139/ssrn.3606770 32697943 PMC7367032

[110] ECDC. 2020 EC for DP and C (ECDC). Q & A on Novel Coronavirus. S. European Centre for Disease Prevention and Control (ECDC). Q & A on Novel Coronavirus. Stockholm: ECDC (2020). Available at: https://www.ecdc.europa.eu/en/covid-19/questions-answers.

[111] He W Yi GY Zhu Y. Estimation of the Basic Reproduction Number, Average Incubation Time, Asymptomatic Infection Rate, and Case Fatality Rate for COVID-19: Meta-Analysis and Sensitivity Analysis. J Med Virol (2020) 92(11):2543–50. doi: 10.1002/jmv.26041 PMC7283745 32470164

[112] Zhou X Ye Q. Cellular Immune Response to COVID-19 and Potential Immune Modulators. Front Immunol (2021) 12. doi: 10.3389/fimmu.2021.646333 PMC8121250 33995364

[113] Huang C Wang Y Li X Ren L Zhao J Hu Y. Clinical Features of Patients Infected With 2019 Novel Coronavirus in Wuhan, China. Lancet (2020) 395(10223):497–506. doi: 10.1016/S0140-6736(20)30183-5 31986264 PMC7159299

[114] Mathew D Giles JR Baxter AE Oldridge DA Greenplate AR Wu JE. Deep Immune Profiling of COVID-19 Patients Reveals Distinct Immunotypes With Therapeutic Implications. Science (2020) 369(6508):eabc8511. doi: 10.1126/science.abc8511 32669297 PMC7402624

[115] Giamarellos-Bourboulis EJ Netea MG Rovina N Akinosoglou K Antoniadou A Antonakos N. Complex Immune Dysregulation in COVID-19 Patients With Severe Respiratory Failure. Cell Host Microbe (2020) 27(6):992–1000.e3. doi: 10.1016/j.chom.2020.04.009 32320677 PMC7172841

[116] Zhou Z Ren L Zhang L Zhong J Xiao Y Jia Z. Heightened Innate Immune Responses in the Respiratory Tract of COVID-19 Patients. Cell Host Microbe (2020) 27(6):883–90.e2. doi: 10.1016/j.chom.2020.04.017 32407669 PMC7196896

[117] Reynolds CJ Swadling L Gibbons JM Pade C Jensen MP Diniz MO. Discordant Neutralizing Antibody and T Cell Responses in Asymptomatic and Mild SARS-CoV-2 Infection. Sci Immunol (2020) 5(54):eabf3698. doi: 10.1126/sciimmunol.abf3698 33361161 PMC8101131

[118] Zuo J Dowell AC Pearce H Verma K Long HM Begum J. Robust SARS-CoV-2-Specific T Cell Immunity is Maintained at 6 Months Following Primary Infection. Nat Immunol (2021) 22(5):620–6. doi: 10.1038/s41590-021-00902-8 PMC7610739 33674800

[119] Zhou R To KKW Wong YC Liu L Zhou B Li X. Acute SARS-CoV-2 Infection Impairs Dendritic Cell and T Cell Responses. Immunity (2020) 53(4):864–77.e5. doi: 10.1016/j.immuni.2020.07.026 32791036 PMC7402670

[120] Liao L Yang Gh. Clinical Significance of Cellular Immunity Function and Inflammatory Factors Assays in Alveolar Lavage Fluid for Severe COVID-19 Pneumonia. J Med Virol (2021) 93(5):2979–87. doi: 10.1002/jmv.26827 PMC8013331 33506950

[121] Gil-Manso S Carbonell D López-Fernández L Miguens I Alonso R Buño I. Induction of High Levels of Specific Humoral and Cellular Responses to SARS-CoV-2 After the Administration of Covid-19 mRNA Vaccines Requires Several Days. Front Immunol (2021) 12. doi: 10.3389/fimmu.2021.726960 PMC8521189 34671348

[122] Domingo P Mur I Pomar V Corominas H Casademont J de Benito N. The Four Horsemen of a Viral Apocalypse: The Pathogenesis of SARS-CoV-2 Infection (COVID-19). EBioMedicine (2020) 58:102887. doi: 10.1016/j.ebiom.2020.102887 32736307 PMC7387269

[123] Baumgarth N Nikolich-Žugich J Lee FE-H Bhattacharya D. Antibody Responses to SARS-CoV-2: Let’s Stick to Known Knowns. J Immunol (2020) 205(9):2342–50. doi: 10.4049/jimmunol.2000839 PMC7578055 32887754

[124] Rydyznski Moderbacher C Ramirez SI Dan JM Grifoni A Hastie KM Weiskopf D. Antigen-Specific Adaptive Immunity to SARS-CoV-2 in Acute COVID-19 and Associations With Age and Disease Severity. Cell (2020) 183(4):996–1012.e19. doi: 10.1016/j.cell.2020.09.038 33010815 PMC7494270

[125] Tan AT Linster M Tan CW Le Bert N Chia WN Kunasegaran K. Early Induction of Functional SARS-CoV-2-Specific T Cells Associates With Rapid Viral Clearance and Mild Disease in COVID-19 Patients. Cell Rep (2021) 34(6):108728. doi: 10.1016/j.celrep.2021.108728 33516277 PMC7826084

[126] Sekine T Perez-Potti A Rivera-Ballesteros O Strålin K Gorin J-B Olsson A. Cell Immunity in Convalescent Individuals with Asymptomatic or Mild COVID-19. Cell (2020) 183(1):158–68.e14. doi: 10.1016/j.cell.2020.08.017 32979941 PMC7427556

[127] Piccoli L Park YJ Tortorici MA Czudnochowski N Walls AC Beltramello M. Mapping Neutralizing and Immunodominant Sites on the SARS-CoV-2 Spike Receptor-Binding Domain by Structure-Guided High-Resolution Serology. Cell (2020) 183(4):1024–42.e21. doi: 10.1016/j.cell.2020.09.037 32991844 PMC7494283

[128] Robbiani DF Gaebler C Muecksch F Lorenzi JCC Wang Z Cho A. Convergent Antibody Responses to SARS-CoV-2 in Convalescent Individuals. Nature (2020) 584(7821):437–42. doi: 10.1038/s41586-020-2456-9 PMC7442695 32555388

[129] Liu A Li Y Peng J Huang Y Xu D. Antibody Responses Against SARS-CoV-2 in COVID-19 Patients. J Med Virol (2021) 93(1):144–8. doi: 10.1002/jmv.26241 PMC7362084 32603501

[130] Soresina A Moratto D Chiarini M Paolillo C Baresi G Focà E. Two X-Linked Agammaglobulinemia Patients Develop Pneumonia as COVID-19 Manifestation But Recover. Pediatr Allergy Immunol (2020) 31(5):565–9. doi: 10.1111/pai.13263 PMC7264678 32319118

[131] Quinti I Lougaris V Milito C Cinetto F Pecoraro A Mezzaroma I. A Possible Role for B Cells in COVID-19? Lesson From Patients With Agammaglobulinemia. J Allergy Clin Immunol (2020) 146(1):211–13.e4. doi: 10.1016/j.jaci.2020.04.013 32333914 PMC7175894

[132] Montero-Escribano P Matías-Guiu J Gómez-Iglesias P Porta-Etessam J Pytel V Matias-Guiu JA. Anti-CD20 and COVID-19 in Multiple Sclerosis and Related Disorders: A Case Series of 60 Patients From Madrid, Spain. Mult Scler Relat Disord (2020) 42:102185. doi: 10.1016/j.msard.2020.102185 32408147 PMC7204643

[133] Novi G Mikulska M Briano F Toscanini F Tazza F Uccelli A. COVID-19 in a MS Patient Treated With Ocrelizumab: Does Immunosuppression Have a Protective Role? Mult Scler Relat Disord (2020) 42:102120. doi: 10.1016/j.msard.2020.102120 32315980 PMC7156942

[134] Safavi F Nourbakhsh B Azimi AR. B-Cell Depleting Therapies May Affect Susceptibility to Acute Respiratory Illness Among Patients With Multiple Sclerosis During the Early COVID-19 Epidemic in Iran. Mult Scler Relat Disord (2020) 43:102195. doi: 10.1016/j.msard.2020.102195 32460086 PMC7219389

[135] Creed MA Ballesteros E Greenfield LJ Jr Imitola J. Mild COVID-19 Infection Despite Chronic B Cell Depletion in a Patient With Aquaporin-4-Positive Neuromyelitis Optica Spectrum Disorder. Mult Scler Relat Disord (2020) 44:102199. doi: 10.1016/j.msard.2020.102199 32554285 PMC7236713

[136] Avouac J Airó P Carlier N Matucci-Cerinic M Allanore Y. Severe COVID-19-Associated Pneumonia in 3 Patients With Systemic Sclerosis Treated With Rituximab. Ann Rheum Dis (2020) annrheumdis-2020-217864. doi: 10.1136/annrheumdis-2020-217864 32503849

[137] Bange EM Han NA Wileyto P Kim JY Gouma S Robinson J. CD8+ T Cells Contribute to Survival in Patients With COVID-19 and Hematologic Cancer. Nat Med (2021) 27(7):1280–9. doi: 10.1038/s41591-021-01386-7 PMC8291091 34017137

[138] Annunziato F Romagnani C Romagnani S. The 3 Major Types of Innate and Adaptive Cell-Mediated Effector Immunity. J Allergy Clin Immunol (2015) 135(3):626–35. doi: 10.1016/j.jaci.2014.11.001 25528359

[139] Iwasaki A Medzhitov R. Control of Adaptive Immunity by the Innate Immune System. Nat Immunol (2015) 16(4):343–53. doi: 10.1038/ni.3123 PMC4507498 25789684

[140] O’Shea J Paul WE. Mechanisms Underlying Lineage Commitment and Plasticity of Helper CD4 + T Cells. Science (2010) 327(5969):1098–102. doi: 10.1126/science.1178334 PMC2997673 20185720

[141] Chen G Wu D Guo W Cao Y Huang D Wang H. Clinical and Immunological Features of Severe and Moderate Coronavirus Disease 2019. J Clin Invest (2020) 130(5):2620–9. doi: 10.1172/JCI137244 PMC7190990 32217835

[142] Lucas C Wong P Klein J Castro TBR Silva J Sundaram M. Longitudinal Analyses Reveal Immunological Misfiring in Severe COVID-19. Nature (2020) 584(7821):463–9. doi: 10.1038/s41586-020-2588-y PMC7477538 32717743

[143] GeurtsvanKessel CH Geers D Schmitz KS Mykytyn AZ Lamers MM Bogers S. Divergent SARS CoV-2 Omicron-Specific T- and B-Cell Responses in COVID-19 Vaccine Recipients. Sci Immunol (2022) 7(69):eabo2202. doi: 10.1126/sciimmunol.abo2202 35113647 PMC8939771

[144] Peng Y Mentzer AJ Liu G Yao X Yin Z Dong D. Broad and Strong Memory CD4+ and CD8+ T Cells Induced by SARS-CoV-2 in UK Convalescent Individuals Following COVID-19. Nat Immunol (2020) 21(11):1336–45. doi: 10.1038/s41590-020-0782-6 PMC7611020 32887977

[145] Kuri-Cervantes L Pampena MB Meng W Rosenfeld AM Ittner CAG Weisman AR. Comprehensive Mapping of Immune Perturbations Associated With Severe COVID-19. Sci Immunol (2020) 5(49):eabd7114. doi: 10.1126/sciimmunol.abd7114 32669287 PMC7402634

[146] Wen W Su W Tang H Le W Zhang X Zheng Y. Immune Cell Profiling of COVID-19 Patients in the Recovery Stage by Single-Cell Sequencing. Cell Discov (2020) 6(1):31. doi: 10.1038/s41421-020-00187-5 32377375 PMC7197635

[147] Liao M Liu Y Yuan J Wen Y Xu G Zhao J. Single-Cell Landscape of Bronchoalveolar Immune Cells in Patients With COVID-19. Nat Med (2020) 26(6):842–4. doi: 10.1038/s41591-020-0901-9 32398875

[148] Ripperger TJ Bhattacharya D. Transcriptional and Metabolic Control of Memory B Cells and Plasma Cells. Annu Rev Immunol (2021) 39:345–68. doi: 10.1146/annurev-immunol-093019-125603 33556247

[149] Victora GD Nussenzweig MC. Germinal Centers. Annu Rev Immunol (2012) 30:429–57. doi: 10.1146/annurev-immunol-020711-075032 22224772

[150] Crotty S. Follicular Helper CD4 T Cells (T Fh). Annu Rev Immunol (2011) 29:621–63. doi: 10.1146/annurev-immunol-031210-101400 21314428

[151] Ueno H Banchereau J Vinuesa CG. Pathophysiology of T Follicular Helper Cells in Humans and Mice. Nat Immunol (2015) 16(2):142–52. doi: 10.1038/ni.3054 PMC4459756 25594465

[152] Turner JS O’Halloran JA Kalaidina E Kim W Schmitz AJ Zhou JQ. SARS-CoV-2 mRNA Vaccines Induce Persistent Human Germinal Centre Responses. Nature (2021) 596(7870):109–13. doi: 10.1038/s41586-021-03738-2 PMC8935394 34182569

[153] Turner JS Zhou JQ Han J Schmitz AJ Rizk AA Alsoussi WB. Human Germinal Centres Engage Memory and Naive B Cells After Influenza Vaccination. Nature (2020) 586(7827):127–32. doi: 10.1038/s41586-020-2711-0 PMC7566073 32866963

[154] Kim W Zhou JQ Sturtz AJ Horvath SC Schmitz AJ Lei T. Germinal Centre-Driven Maturation of B Cell Response to SARS-CoV-2 Vaccination. bioRxiv (2021) 2:2021.10.31.466651. doi: 10.1101/2021.10.31.466651

[155] Mudd PA Minervina AA Pogorelyy MV Turner JS Kim W Kalaidina E. SARS-CoV-2 mRNA Vaccination Elicits a Robust and Persistent T Follicular Helper Cell Response in Humans. Cell (2022) 185(4):603–13.e15. doi: 10.1016/j.cell.2021 35026152 PMC8695127

[156] Touizer E Alrubayyi A Rees-Spear C Fisher-Pearson N Griffith SA Muir L. Failure to Seroconvert After Two Doses of BNT162b2 SARS-CoV-2 Vaccine in a Patient With Uncontrolled HIV. Lancet HIV (2021) 8(6):e317–8. doi: 10.1016/S2352-3018(21)00099-0 PMC8169058 34087093

[157] Kamar N Abravanel F Marion O Couat C Izopet J Del Bello A. Three Doses of an mRNA Covid-19 Vaccine in Solid-Organ Transplant Recipients. N Engl J Med (2021) 385(7):661–2. doi: 10.1056/NEJMc2108861 PMC8262620 34161700

[158] Kaneko N Kuo HH Boucau J Farmer JR Allard-Chamard H Mahajan VS. Loss of Bcl-6-Expressing T Follicular Helper Cells and Germinal Centers in COVID-19. Cell (2020) 183(1):143–57.e13. doi: 10.1016/j.cell.2020.08.025 32877699 PMC7437499

[159] Magleby R Westblade LF Trzebucki A Simon MS Rajan M Park J. Impact of Severe Acute Respiratory Syndrome Coronavirus 2 Viral Load on Risk of Intubation and Mortality Among Hospitalized Patients With Coronavirus Disease 2019. Clin Infect Dis (2021) 73(11):e4197–205. doi: 10.1093/cid/ciaa851 PMC7337625 32603425

[160] Zheng M Gao Y Wang G Song G Liu S Sun D. Functional Exhaustion of Antiviral Lymphocytes in COVID-19 Patients. Cell Mol Immunol (2020) 17(5):533–5. doi: 10.1038/s41423-020-0402-2 PMC7091858 32203188

[161] McMahan K Yu J Mercado NB Loos C Tostanoski LH Chandrashekar A. Correlates of Protection Against SARS-CoV-2 in Rhesus Macaques. Nature (2021) 590(7847):630–4. doi: 10.1038/s41586-020-03041-6 PMC7906955 33276369

[162] Wyllie DH Mulchandani R Jones HE Taylor-Phillips S Brooks T Charlett A. Responsive T Cell Numbers Are Associated With Protection From COVID-19: A Prospective Cohort Study in Keyworkers. medRxiv (2020) 04:2020.11.02.20222778. doi: 10.1101/2020.11.02.20222778.

[163] Ni L Cheng ML Feng Y Zhao H Liu J Ye F. Impaired Cellular Immunity to SARS-CoV-2 in Severe COVID-19 Patients. Front Immunol (2021) 12. doi: 10.3389/fimmu.2021.603563 PMC7884325 33603759

[164] Xu Z Shi L Wang Y Zhang J Huang L Zhang C. Pathological Findings of COVID-19 Associated With Acute Respiratory Distress Syndrome. Lancet Respir Med (2020) 8(4):420–2. doi: 10.1016/S2213-2600(20)30076-X PMC7164771 32085846

[165] Diao B Wang C Tan Y Chen X Liu Y Ning L. Reduction and Functional Exhaustion of T Cells in Patients With Coronavirus Disease 2019 (COVID-19). Front Immunol (2020) 11. doi: 10.3389/fimmu.2020.00827 PMC7205903 32425950

[166] Billerbeck E Kang YH Walker L Lockstone H Grafmueller S Fleming V. Analysis of CD161 Expression on Human CD8+ T Cells Defines a Distinct Functional Subset With Tissue-Homing Properties. Proc Natl Acad Sci USA (2010) 107(7):3006–11. doi: 10.1073/pnas.0914839107 PMC2840308 20133607

[167] Van Wilgenburg B Scherwitzl I Hutchinson EC Leng T Kurioka A Kulicke C. MAIT Cells are Activated During Human Viral Infections. Nat Commun (2016) 7:11653. doi: 10.1038/ncomms11653 27337592 PMC4931007

[168] Jouan Y Guillon A Gonzalez L Perez Y Boisseau C Ehrmann S. Phenotypical and Functional Alteration of Unconventional T Cells in Severe COVID-19 Patients. J Exp Med (2020) 217(12):e20200872. doi: 10.1084/jem.20200872 32886755 PMC7472174

[169] Blanchard-Rohner G Pulickal AS Jol-van Der Zijde CM Snape MD Pollard AJ. Appearance of Peripheral Blood Plasma Cells and Memory B Cells in a Primary and Secondary Immune Response in Humans. Blood (2009) 114(24):4998–5002. doi: 10.1182/blood-2009-03-211052 19843885 PMC2788974

[170] Lee FE-H Halliley JL Walsh EE Moscatiello AP Kmush BL Falsey AR. Circulating Human Antibody-Secreting Cells During Vaccinations and Respiratory Viral Infections Are Characterized by High Specificity and Lack of Bystander Effect. J Immunol (2011) 186(9):5514–21. doi: 10.4049/jimmunol.1002932 PMC3726212 21441455

[171] Wrammert J Onlamoon N Akondy RS Perng GC Polsrila K Chandele A. Rapid and Massive Virus-Specific Plasmablast Responses During Acute Dengue Virus Infection in Humans. J Virol (2012) 86(6):2911–8. doi: 10.1128/JVI.06075-11 PMC3302324 22238318

[172] Moore JB June CH. Cytokine Release Syndrome in Severe COVID-19. Sci (80- ) (2020) 368(6490):473–4. doi: 10.1126/science.abb8925 32303591

[173] Tay MZ Poh CM Rénia L MacAry PA Ng LFP. The Trinity of COVID-19: Immunity, Inflammation and Intervention. Nat Rev Immunol (2020) 20(6):363–74. doi: 10.1038/s41577-020-0311-8 PMC7187672 32346093

[174] Guo XzJ Thomas PG. New Fronts Emerge in the Influenza Cytokine Storm. Semin Immunopathol (2017) 39(5):541–50. doi: 10.1007/s00281-017-0636-y PMC5580809 28555383

[175] Wan S Yi Q Fan S Lv J Zhang X Guo L. Relationships Among Lymphocyte Subsets, Cytokines, and the Pulmonary Inflammation Index in Coronavirus (COVID-19) Infected Patients. Br J Haematol (2020) 189(3):428–37. doi: 10.1111/bjh.16659 PMC7262036 32297671

[176] Lee JS Park S Jeong HW Ahn JY Choi SJ Lee H. Immunophenotyping of Covid-19 and Influenza Highlights the Role of Type I Interferons in Development of Severe Covid-19. Sci Immunol (2020) 5(49):eabd1554. doi: 10.1126/sciimmunol.abd1554 32651212 PMC7402635

[177] Zhang Q Liu Z Moncada-Velez M Chen J Ogishi M Bigio B. Inborn Errors of Type I IFN Immunity in Patients With Life-Threatening COVID-19. Science (2020) 370(6515):eabd4570. doi: 10.1126/science.abd4570 32972995 PMC7857407

[178] Blanco-Melo D Nilsson-Payant BE Liu WC Uhl S Hoagland D Møller R. Imbalanced Host Response to SARS-CoV-2 Drives Development of COVID-19. Cell (2020) 181(5):1036–45.e9. doi: 10.1016/j.cell.2020.04.026 32416070 PMC7227586

[179] Ferrucci L Fabbri E. Inflammageing: Chronic Inflammation in Ageing, Cardiovascular Disease, and Frailty. Nat Rev Cardiol (2018) 15(9):505–22. doi: 10.1038/s41569-018-0064-2 PMC6146930 30065258

[180] Shaw AC Goldstein DR Montgomery RR. Age-Dependent Dysregulation of Innate Immunity. Nat Rev Immunol (2013) 13(12):875–87. doi: 10.1038/nri3547 PMC4096436 24157572

[181] Qin C Zhou L Hu Z Zhang S Yang S Tao Y. Dysregulation of Immune Response in Patients With Coronavirus 2019 (COVID-19) in Wuhan, China. Clin Infect Dis (2020) 71(15):762–8. doi: 10.1093/cid/ciaa248 PMC7108125 32161940

[182] Thieme CJ Anft M Paniskaki K Blazquez-Navarro A Doevelaar A Seibert FS. Robust T Cell Response Toward Spike, Membrane, and Nucleocapsid SARS-CoV-2 Proteins Is Not Associated With Recovery in Critical COVID-19 Patients. Cell Rep Med (2020) 1(6):100092. doi: 10.1016/j.xcrm.2020.100092 32904468 PMC7456276

[183] Vaz de Paula CB de Azevedo MLV Nagashima S Martins APC Malaquias MAS Miggiolaro A. IL-4/IL-13 Remodeling Pathway of COVID-19 Lung Injury. Sci Rep (2020) 10(1):18689. doi: 10.1038/s41598-020-75659-5 33122784 PMC7596721

[184] Melgaço JG Brito E Cunha D Azamor T Da Silva AMV Tubarão LN Gonçalves RB. Cellular and Molecular Immunology Approaches for the Development of Immunotherapies Against the New Coronavirus (SARS-CoV-2): Challenges to Near-Future Breakthroughs. J Immunol Res (2020) 2020:8827670. doi: 10.1155/2020/8827670 33426096 PMC7753942

[185] Martonik D Parfieniuk-Kowerda A Rogalska M Flisiak R. The Role of Th17 Response in COVID-19. Cells (2021) 10(6):1550. doi: 10.3390/cells10061550 34205262 PMC8235311

[186] Schulte-Schrepping J Reusch N Paclik D Baßler K Schlickeiser S Zhang B. Severe COVID-19 Is Marked by a Dysregulated Myeloid Cell Compartment. Cell (2020) 182(6):1419–40.e23. doi: 10.1016/j.cell.2020.08.001 32810438 PMC7405822

[187] Deng Z Zhang M Zhu T Zhili N Liu Z Xiang R. Dynamic Changes in Peripheral Blood Lymphocyte Subsets in Adult Patients With COVID-19. Int J Infect Dis (2020) 98:353–8. doi: 10.1016/j.ijid.2020.07.003 PMC7334931 32634585

[188] Wherry EJ. T Cell Exhaustion. Nat Immunol (2011) 12(6):492–9. doi: 10.1038/ni.2035 21739672

[189] Brooks DG Trifilo MJ Edelmann KH Teyton L McGavern DB Oldstone MBA. Interleukin-10 Determines Viral Clearance or Persistence In Vivo. Nat Med (2006) 12(11):1301–9. doi: 10.1038/nm1492 PMC2535582 17041596

[190] Zheng HY Zhang M Yang CX Zhang N Wang XC Yang XP. Elevated Exhaustion Levels and Reduced Functional Diversity of T Cells in Peripheral Blood may Predict Severe Progression in COVID-19 Patients. Cell Mol Immunol (2020) 17(5):541–3. doi: 10.1038/s41423-020-0401-3 PMC7091621 32203186

[191] Kreutmair S Unger S Núñez NG Ingelfinger F Alberti C De Feo D. Distinct Immunological Signatures Discriminate Severe COVID-19 From non-SARS-CoV-2-Driven Critical Pneumonia. Immunity (2021) 54(7):1578–93.e5. doi: 10.1016/j.immuni.2021.05.002 34051147 PMC8106882

[192] Rha MS Jeong HW Ko JH Choi SJ Seo IH Lee JS. PD-1-Expressing SARS-CoV-2-Specific CD8+ T Cells Are Not Exhausted, But Functional in Patients With COVID-19. Immunity (2021) 54(1):44–52.e3. doi: 10.1016/j.immuni.2020.12.002 33338412 PMC7834198

[193] Doering TA Crawford A Angelosanto JM Paley MA Ziegler CG Wherry EJ. Network Analysis Reveals Centrally Connected Genes and Pathways Involved in CD8+ T Cell Exhaustion Versus Memory. Immunity (2012) 37(6):1130–44. doi: 10.1016/j.immuni.2012.08.021 PMC3749234 23159438

[194] Fuertes Marraco SA Neubert NJ Verdeil G Speiser DE. Inhibitory Receptors Beyond T Cell Exhaustion. Front Immunol (2015) 6(JUN). doi: 10.3389/fimmu.2015.00310 PMC4481276 26167163

[195] Singer M Wang C Cong L Marjanovic ND Kowalczyk MS Zhang H. A Distinct Gene Module for Dysfunction Uncoupled From Activation in Tumor-Infiltrating T Cells. Cell (2016) 166(6):1500–11.e9. doi: 10.1016/j.cell.2016.08.052 27610572 PMC5019125

[196] Tirosh I Izar B Prakadan SM Wadsworth MH Treacy D Trombetta JJ. Dissecting the Multicellular Ecosystem of Metastatic Melanoma by Single-Cell RNA-Seq. Sci (2016) 352(6282):189–96. doi: 10.1126/science.aad0501 PMC4944528 27124452

[197] Youngblood B Oestreich KJ Ha SJ Duraiswamy J Akondy RS West EE. Chronic Virus Infection Enforces Demethylation of the Locus That Encodes PD-1 in Antigen-Specific CD8+ T Cells. Immunity (2011) 35(3):400–12. doi: 10.1016/j.immuni.2011.06.015 PMC3183460 21943489

[198] Saris A Reijnders TDY Nossent EJ Schuurman AR Verhoeff J Van Asten S. Distinct Cellular Immune Profiles in the Airways and Blood of Critically Ill Patients With COVID-19. Thorax (2021) 76(10):1010–9. doi: 10.1136/thoraxjnl-2020-216256 PMC8050882 33846275

[199] Vitte J Diallo AB Boumaza A Lopez A Michel M Allardet-Servent J. A Granulocytic Signature Identifies COVID-19 and its Severity. J Infect Dis (2020) 222(12):1985–96. doi: 10.1093/infdis/jiaa591 PMC7543529 32941618

[200] Chen J Vitetta L. Increased PD-L1 Expression may be Associated With the Cytokine Storm and CD8+T-Cell Exhaustion in Severe COVID-19. J Infect Dis (2021) 223(9):1659–60. doi: 10.1093/infdis/jiab061 PMC7928766 33524110

[201] Sabbatino F Conti V Franci G Sellitto C Manzo V Pagliano P. PD-L1 Dysregulation in COVID-19 Patients. Front Immunol (2021) 12. doi: 10.3389/fimmu.2021.695242 PMC8215357 34163490

[202] Eljaafari A Pestel J Le Magueresse-Battistoni B Chanon S Watson J Robert M. Adipose-Tissue-Derived Mesenchymal Stem Cells Mediate PD-L1 Overexpression in the White Adipose Tissue of Obese Individuals, Resulting in T Cell Dysfunction. Cells (2021) 10(10):2645. doi: 10.3390/cells10102645 34685625 PMC8534339

[203] Dorward DA Russell CD Um IH Elshani M Armstrong SD Penrice-Randal R. Tissue-Specific Immunopathology in Fatal COVID-19. Am J Respir Crit Care Med (2021) 203(2):192–201. doi: 10.1164/rccm.202008-3265OC 33217246 PMC7874430

[204] Liu J Yang X Wang H Li Z Deng H Liu J. Analysis of the Long-Term Impact on Cellular Immunity in COVID-19-Recovered Individuals Reveals a Profound Nkt Cell Impairment. MBio (2021) 12(2):e00085–21. doi: 10.1128/mBio.00085-21 33906918 PMC8092197

[205] Aid M Busman-Sahay K Vidal SJ Maliga Z Bondoc S Starke C. Vascular Disease and Thrombosis in SARS-CoV-2-Infected Rhesus Macaques. Cell (2020) 183(5):1354–66.e13. doi: 10.1016/j.cell.2020.10.005 33065030 PMC7546181

[206] Li S Jiang L Li X Lin F Wang Y Li B. Clinical and Pathological Investigation of Patients With Severe COVID-19. JCI Insight (2020) 5(12):e138070. doi: 10.1172/jci.insight.138070 PMC7406259 32427582

[207] Radermecker C Detrembleur N Guiot J Cavalier E Henket M d’Emal C. Neutrophil Extracellular Traps Infiltrate the Lung Airway, Interstitial, and Vascular Compartments in Severe COVID-19. J Exp Med (2020) 217(12):e20201012. doi: 10.1084/jem.20201012 32926097 PMC7488867

[208] Schurink B Roos E Radonic T Barbe E Bouman CSC de Boer HH. Viral Presence and Immunopathology in Patients With Lethal COVID-19: A Prospective Autopsy Cohort Study. Lancet Microbe (2020) 1(7):e290–9. doi: 10.1016/S2666-5247(20)30144-0 PMC7518879 33015653

[209] Del Valle DM Kim-Schulze S Huang HH Beckmann ND Nirenberg S Wang B. An Inflammatory Cytokine Signature Predicts COVID-19 Severity and Survival. Nat Med (2020) 26(10):1636–43. doi: 10.1038/s41591-020-1051-9 PMC7869028 32839624

[210] Takahashi T Ellingson MK Wong P Israelow B Lucas C Klein J. Sex Differences in Immune Responses That Underlie COVID-19 Disease Outcomes. Nature (2020) 588(7837):315–20. doi: 10.1038/s41586-020-2700-3 PMC7725931 32846427

[211] Li C Lee A Grigoryan L Arunachalam PS Scott MKD Trisal M. Mechanisms of Innate and Adaptive Immunity to the Pfizer-BioNTech BNT162b2 Vaccine. Nat Immunol (2022) 23:543–555. doi: 10.1038/s41590-022-01163-9 35288714 PMC8989677

[212] Loske J Röhmel J Lukassen S Stricker S Magalhães VG Liebig J. Pre-Activated Antiviral Innate Immunity in the Upper Airways Controls Early SARS-CoV-2 Infection in Children. Nat Biotechnol (2021) 40:319–24. doi: 10.1101/2021.06.24.21259087 34408314

[213] Dufort EM Koumans EH Chow EJ Rosenthal EM Muse A Rowlands J. Multisystem Inflammatory Syndrome in Children in New York State. N Engl J Med (2020) 383(4):347–58. doi: 10.1056/NEJMoa2021756 PMC7346766 32598830

[214] Feldstein LR Rose EB Horwitz SM Collins JP Newhams MM Son MBF. Multisystem Inflammatory Syndrome in U.S. Children and Adolescents. N Engl J Med (2020) 383(4):334–46. doi: 10.1056/NEJMoa2021680 PMC7346765 32598831

[215] Weisberg SP Connors TJ Zhu Y Baldwin MR Lin WH Wontakal S. Distinct Antibody Responses to SARS-CoV-2 in Children and Adults Across the COVID-19 Clinical Spectrum. Nat Immunol (2021) 22(1):25–31. doi: 10.1038/s41590-020-00826-9 33154590 PMC8136619

[216] Whittaker E Bamford A Kenny J Kaforou M Jones CE Shah P. Clinical Characteristics of 58 Children With a Pediatric Inflammatory Multisystem Syndrome Temporally Associated With SARS-CoV-2. JAMA - J Am Med Assoc (2020) 324(3):259–69. doi: 10.1001/jama.2020.10369 PMC7281356 32511692

[217] Zhang J Lin H Ye B Zhao M Zhan J Dong S. One-Year Sustained Cellular and Humoral Immunities of COVID-19 Convalescents. Clin Infect Dis (2021) ciab884. doi: 10.1093/cid/ciab884 34609506 PMC8524303

[218] Dan JM Mateus J Kato Y Hastie KM Yu ED Faliti CE. Immunological Memory to SARS-CoV-2 Assessed for Up to 8 Months After Infection. Science (2021) 371(6529):eabf4063. doi: 10.1126/science.abf4063 33408181 PMC7919858

[219] Bilich T Nelde A Heitmann JS Maringer Y Roerden M Bauer J. T Cell and Antibody Kinetics Delineate SARS-CoV-2 Peptides Mediating Long-Term Immune Responses in COVID-19 Convalescent Individuals. Sci Transl Med (2021) 13(590):eabf7517. doi: 10.1126/scitranslmed.abf7517 33723016 PMC8128286

[220] He Z Ren L Yang J Guo L Feng L Ma C. Seroprevalence and Humoral Immune Durability of Anti-SARS-CoV-2 Antibodies in Wuhan, China: A Longitudinal, Population-Level, Cross-Sectional Study. Lancet (2021) 397(10279):1075–84. doi: 10.1016/S0140-6736(21)00238-5 PMC7972311 33743869

[221] Gurevich M Zilkha-Falb R Sonis P Magalashvili D Menascu S Flechter S. SARS-CoV-2 Memory B and T Cell Profiles in Mild COVID-19 Convalescent Patients. Int J Infect Dis (2022) 115:208–14. doi: 10.1016/j.ijid.2021.12.309 PMC8653411 34896265

[222] Bonifacius A Tischer-Zimmermann S Dragon AC Gussarow D Vogel A Krettek U. COVID-19 Immune Signatures Reveal Stable Antiviral T Cell Function Despite Declining Humoral Responses. Immunity (2021) 54(2):340–54.e6. doi: 10.1016/j.immuni.2021.01.008 33567252 PMC7871825

[223] Huang C Huang L Wang Y Li X Ren L Gu X. 6-Month Consequences of COVID-19 in Patients Discharged From Hospital: A Cohort Study. Lancet (2021) 397(10270):220–32. doi: 10.1016/S0140-6736(20)32656-8 PMC7833295 33428867

[224] Wheatley AK Juno JA Wang JJ Selva KJ Reynaldi A Tan HX. Evolution of Immune Responses to SARS-CoV-2 in Mild-Moderate COVID-19. Nat Commun (2021) 12(1):1162. doi: 10.1038/s41467-021-21444-5 33608522 PMC7896046

[225] Sherina N Piralla A Du L Wan H Kumagai-Braesch M Andréll J. Persistence of SARS-CoV-2-Specific B and T Cell Responses in Convalescent COVID-19 Patients 6–8 Months After the Infection. Med (2021) 2(3):281–95.e4. doi: 10.1016/j.medj.2021.02.001 33589885 PMC7874960

[226] Rodda LB Netland J Shehata L Pruner KB Morawski PA Thouvenel CD. Functional SARS-CoV-2-Specific Immune Memory Persists After Mild COVID-19. Cell (2021) 184(1):169–83.e17. doi: 10.1016/j.cell.2020.11.029 33296701 PMC7682481

[227] Jung JH Rha MS Sa M Choi HK Jeon JH Seok H. SARS-CoV-2-Specific T Cell Memory Is Sustained in COVID-19 Convalescent Patients for 10 Months With Successful Development of Stem Cell-Like Memory T Cells. Nat Commun (2021) 12(1):4043. doi: 10.1038/s41467-021-24377-1 34193870 PMC8245549

[228] Grau-Expósito J Sánchez-Gaona N Massana N Suppi M Astorga-Gamaza A Perea D. Peripheral and Lung Resident Memory T Cell Responses Against SARS-CoV-2. Nat Commun (2021) 12(1):3010. doi: 10.1038/s41467-021-23333-3 34021148 PMC8140108

[229] Szabo PA Dogra P Gray JI Wells SB Connors TJ Weisberg SP. Longitudinal Profiling of Respiratory and Systemic Immune Responses Reveals Myeloid Cell-Driven Lung Inflammation in Severe COVID-19. Immunity (2021) 54(4):797–814.e6. doi: 10.1016/j.immuni.2021.03.005 33765436 PMC7951561

[230] Mazzoni A Vanni A Spinicci M Lamacchia G Kiros ST Rocca A. SARS-CoV-2 Infection and Vaccination Trigger Long-Lived B and CD4+ T Lymphocytes: Implications for Booster Strategies. J Clin Invest (2022) 132(6):e157990. doi: 10.1172/JCI157990 35139036 PMC8920339

[231] Vinuesa CG Linterman MA Yu D Maclennan ICM. Follicular Helper T Cells. Annu Rev Immunol (2016) 34:335–68. doi: 10.1146/annurev-immunol-041015-055605 26907215

[232] Juno JA Tan HX Lee WS Reynaldi A Kelly HG Wragg K. Humoral and Circulating Follicular Helper T Cell Responses in Recovered Patients With COVID-19. Nat Med (2020) 26(9):1428–34. doi: 10.1038/s41591-020-0995-0 32661393

[233] Boppana S Qin K Files JK Russell RM Stoltz R Bibollet-Ruche F. SARS-CoV-2-Specific Circulating T Follicular Helper Cells Correlate With Neutralizing Antibodies and Increase During Early Convalescence. PLoS Pathog (2021) 17(7):e1009761. doi: 10.1371/journal.ppat.1009761 34270631 PMC8318272

[234] Stephenson E Reynolds G Botting RA Calero-Nieto FJ Morgan MD Tuong ZK. Single-Cell Multi-Omics Analysis of the Immune Response in COVID-19. Nat Med (2021) 27(5):904–16. doi: 10.1038/s41591-021-01329-2 PMC8121667 33879890

[235] Northfield JW Loo CP Barbour JD Spotts G Hecht FM Klenerman P. Human Immunodeficiency Virus Type 1 (HIV-1)-Specific CD8 + T EMRA Cells in Early Infection Are Linked to Control of HIV-1 Viremia and Predict the Subsequent Viral Load Set Point. J Virol (2007) 81(11):5759–65. doi: 10.1128/JVI.00045-07 PMC1900265 17376902

[236] Lilleri D Fornara C Revello MG Gerna G. Human Cytomegalovirus-Specific Memory CD8+ and CD4+ T Cell Differentiation After Primary Infection. J Infect Dis (2008) 198(4):536–43. doi: 10.1086/590118 18590456

[237] Akondy RS Monson ND Miller JD Edupuganti S Teuwen D Wu H. The Yellow Fever Virus Vaccine Induces a Broad and Polyfunctional Human Memory CD8 + T Cell Response. J Immunol (2009) 183(12):7919–30. doi: 10.4049/jimmunol.0803903 PMC3374958 19933869

[238] Dunne PJ Faint JM Gudgeon NH Fletcher JM Plunkett FJ Soares MVD. Epstein-Barr Virus-Specific CD8+ T Cells That Re-Express CD45RA are Apoptosis-Resistant Memory Cells That Retain Replicative Potential. Blood (2002) 100(3):933–40. doi: 10.1182/blood-2002-01-0160 12130505

[239] Adamo S Michler J Zurbuchen Y Cervia C Taeschler P Raeber ME. Signature of Long-Lived Memory CD8+ T Cells in Acute SARS-CoV-2 Infection. Nature (2022) 602(7895):148–55. doi: 10.1038/s41586-021-04280-x PMC8810382 34875673

[240] Kolumam GA Thomas S Thompson LJ Sprent J Murali-Krishna K. Type I Interferons Act Directly on CD8 T Cells to Allow Clonal Expansion and Memory Formation in Response to Viral Infection. J Exp Med (2005) 202(5):637–50. doi: 10.1084/jem.20050821 PMC2212878 16129706

[241] Derhovanessian E Maier AB Hähnel KH Beck R de Craen AJM Slagboom EP. Infection With Cytomegalovirus But Not Herpes Simplex Virus Induces the Accumulation of Latedifferentiated CD4 + and CD8 + T-Cells in Humans. J Gen Virol (2011) 92(12):2746–56. doi: 10.1099/vir.0.036004-0 21813708

[242] Visvabharathy L Hanson B Orban Z Lim PH Palacio N Jain R. Neuro-COVID Long-Haulers Exhibit Broad Dysfunction in T Cell Memory Generation and Responses to Vaccination. medRxiv (2021) 29:2021.08.08.21261763. doi: 10.1101/2021.08.08.21261763

[243] Gattinoni L Speiser DE Lichterfeld M Bonini C. T Memory Stem Cells in Health and Disease. Nat Med (2017) 23(1):18–27. doi: 10.1038/nm.4241 28060797 PMC6354775

[244] Gattinoni L Lugli E Ji Y Pos Z Paulos CM Quigley MF. A Human Memory T Cell Subset With Stem Cell-Like Properties. Nat Med (2011) 17(10):1290–7. doi: 10.1038/nm.2446 PMC3192229 21926977

[245] Cohen KW Linderman SL Moodie Z Czartoski J Lai L Mantus G. Longitudinal Analysis Shows Durable and Broad Immune Memory After SARS-CoV-2 Infection With Persisting Antibody Responses and Memory B and T Cells. Cell Rep Med (2021) 2(7):100354. doi: 10.1101/2021.04.19.21255739 34250512 PMC8253687

[246] Forthal D. Adaptive Immune Responses to SARS-CoV-2. Adv Drug Delivery Rev (2021) 172:1–8. doi: 10.1016/j.addr.2021.02.009 PMC7891074 33610693

[247] Galletti G De Simone G Mazza EMC Puccio S Mezzanotte C Bi TM. Two Subsets of Stem-Like CD8+ Memory T Cell Progenitors With Distinct Fate Commitments in Humans. Nat Immunol (2020) 21(12):1552–62. doi: 10.1038/s41590-020-0791-5 PMC7610790 33046887

[248] Cox RJ Brokstad KA. Not Just Antibodies: B Cells and T Cells Mediate Immunity to COVID-19. Nat Rev Immunol (2020) 20(10):581–2. doi: 10.1038/s41577-020-00436-4 PMC7443809 32839569

[249] Mazzoni A Di Lauria N Maggi L Salvati L Vanni A Capone M. First-Dose mRNA Vaccination is Sufficient to Reactivate Immunological Memory to SARS-CoV-2 in Subjects Who Have Recovered From COVID-19. J Clin Invest (2021) 131(12):e149150. doi: 10.1172/JCI149150 PMC8203460 33939647

[250] Painter MM Mathew D Goel RR Apostolidis SA Pattekar A Kuthuru O. Rapid Induction of Antigen-Specific CD4+ T Cells is Associated With Coordinated Humoral and Cellular Immunity to SARS-CoV-2 mRNA Vaccination. Immunity (2021) 54(9):2133–42.e3. doi: 10.1016/j.immuni.2021.08.001 34453880 PMC8361141

[251] Polack FP Thomas SJ Kitchin N Absalon J Gurtman A Lockhart S. Safety and Efficacy of the BNT162b2 mRNA Covid-19 Vaccine. N Engl J Med (2020) 383(27):2603–15. doi: 10.1056/NEJMoa2034577 PMC7745181 33301246

[252] Kalimuddin S Tham CYL Qui M de Alwis R Sim JXY Lim JME. Early T Cell and Binding Antibody Responses are Associated With COVID-19 RNA Vaccine Efficacy Onset. Med (2021) 2(6):682–88.e4. doi: 10.1016/j.medj.2021.04.003 33851143 PMC8030737

[253] Sahin U Muik A Derhovanessian E Vogler I Kranz LM Vormehr M. COVID-19 Vaccine BNT162b1 Elicits Human Antibody and TH1 T Cell Responses. Nature (2020) 586(7830):594–9. doi: 10.1038/s41586-020-2814-7 32998157

[254] Sahin U Muik A Vogler I Derhovanessian E Kranz LM Vormehr M. BNT162b2 Vaccine Induces Neutralizing Antibodies and Poly-Specific T Cells in Humans. Nature (2021) 595(7868):572–7. doi: 10.1038/s41586-021-03653-6 34044428

[255] Collier DA De Marco A Ferreira IATM Meng B Datir RP Walls AC. Sensitivity of SARS-CoV-2 B.1.1.7 to mRNA Vaccine-Elicited Antibodies. Nature (2021) 593(7857):136–41. doi: 10.1038/s41586-021-03412-7 PMC7616976 33706364

[256] Wang Z Schmidt F Weisblum Y Muecksch F Barnes CO Finkin S. mRNA Vaccine-Elicited Antibodies to SARS-CoV-2 and Circulating Variants. Nature (2021) 592(7855):616–22. doi: 10.1038/s41586-021-03324-6 PMC8503938 33567448

[257] Goel RR Apostolidis SA Painter MM Mathew D Pattekar A Kuthuru O. Distinct Antibody and Memory B Cell Responses in SARSCoV-2 Naïve and Recovered Individuals Following mRNA Vaccination. Sci Immunol (2021) 6(58):1–19. doi: 10.1126/sciimmunol.abi6950 PMC8158969 33858945

[258] Jackson LA Anderson EJ Rouphael NG Roberts PC Makhene M Coler RN. An mRNA Vaccine Against SARS-CoV-2 — Preliminary Report. N Engl J Med (2020) 383(20):1920–31. doi: 10.1056/NEJMoa2022483 PMC7377258 32663912

[259] Saadat S Rikhtegaran Tehrani Z Logue J Newman M Frieman MB Harris AD. Binding and Neutralization Antibody Titers After a Single Vaccine Dose in Health Care Workers Previously Infected With SARS-CoV-2. JAMA - J Am Med Assoc (2021) 325(14):1467–9. doi: 10.1001/jama.2021.3341 PMC7922233 33646292

[260] McDade TW Demonbreun AR Sancilio A Mustanski B D’Aquila RT McNally EM. Durability of Antibody Response to Vaccination and Surrogate Neutralization of Emerging Variants Based on SARS-CoV-2 Exposure History. Sci Rep (2021) 11(1):17325. doi: 10.1038/s41598-021-96879-3 34462501 PMC8405730

[261] Naaber P Tserel L Kangro K Sepp E Jürjenson V Adamson A. Dynamics of Antibody Response to BNT162b2 Vaccine After Six Months: A Longitudinal Prospective Study. Lancet Reg Health Eur (2021) 10:100208. doi: 10.1016/j.lanepe.2021.100208 34514454 PMC8418937

[262] Quast I Tarlinton D. B Cell Memory: Understanding COVID-19. Immunity (2021) 54(2):205–10. doi: 10.1016/j.immuni.2021.01.014 PMC7826135 33513337

[263] Khodadadi L Cheng Q Radbruch A Hiepe F. The Maintenance of Memory Plasma Cells. Front Immunol (2019) 10. doi: 10.3389/fimmu.2019.00721 PMC6464033 31024553

[264] Chivu-Economescu M Bleotu C Grancea C Chiriac D Botezatu A Iancu IV. Kinetics and Persistence of Cellular and Humoral Immune Responses to SARS-CoV-2 Vaccine in Healthcare Workers With or Without Prior COVID-19. J Cell Mol Med (2022) 26(4):1293–305. doi: 10.1111/jcmm.17186 PMC8831971 35043552

[265] Schmidt T Klemis V Schub D Schneitler S Reichert MC Wilkens H. Cellular Immunity Predominates Over Humoral Immunity After Homologous and Heterologous mRNA and Vector-Based COVID-19 Vaccine Regimens in Solid Organ Transplant Recipients. Am J Transplant (2021) 21(12):3990–4002. doi: 10.1111/ajt.16818 34453872 PMC8652989

[266] Zhu J. T Helper 2 (Th2) Cell Differentiation, Type 2 Innate Lymphoid Cell (ILC2) Development and Regulation of Interleukin-4 (IL-4) and IL-13 Production. Cytokine (2015) 75(1):14–24. doi: 10.1016/j.cyto.2015.05.010 26044597 PMC4532589

[267] Bolles M Deming D Long K Agnihothram S Whitmore A Ferris M. A Double-Inactivated Severe Acute Respiratory Syndrome Coronavirus Vaccine Provides Incomplete Protection in Mice and Induces Increased Eosinophilic Proinflammatory Pulmonary Response Upon Challenge. J Virol (2011) 85(23):12201–15. doi: 10.1128/JVI.06048-11 PMC3209347 21937658

[268] Agrawal AS Tao X Algaissi A Garron T Narayanan K Peng BH. Immunization With Inactivated Middle East Respiratory Syndrome Coronavirus Vaccine Leads to Lung Immunopathology on Challenge With Live Virus. Hum Vaccines Immunother (2016) 12(9):2351–6. doi: 10.1080/21645515.2016.1177688 PMC5027702 27269431

[269] Yasui F Kai C Kitabatake M Inoue S Yoneda M Yokochi S. Prior Immunization With Severe Acute Respiratory Syndrome (SARS)-Associated Coronavirus (SARS-CoV) Nucleocapsid Protein Causes Severe Pneumonia in Mice Infected With SARS-CoV. J Immunol (2008) 181(9):6337–48. doi: 10.4049/jimmunol.181.9.6337 18941225

[270] Iwata-Yoshikawa N Uda A Suzuki T Tsunetsugu-Yokota Y Sato Y Morikawa S. Effects of Toll-Like Receptor Stimulation on Eosinophilic Infiltration in Lungs of BALB/c Mice Immunized With UV-Inactivated Severe Acute Respiratory Syndrome-Related Coronavirus Vaccine. J Virol (2014) 88(15):8597–614. doi: 10.1128/JVI.00983-14 PMC4135953 24850731

[271] Xu K Dai L Gao GF. Humoral and Cellular Immunity and the Safety of COVID-19 Vaccines: A Summary of Data Published by 21 May 2021. Int Immunol (2021) 33(10):529–40. doi: 10.1093/intimm/dxab061 PMC8499872 34491327

[272] Krawczyk CM Shen H Pearce EJ. Memory CD4 T Cells Enhance Primary CD8 T-Cell Responses. Infect Immun (2007) 75(7):3556–60. doi: 10.1128/IAI.00086-07 PMC1932926 17438031

[273] Luckheeram RV Zhou R Verma AD Xia B. CD4 +T Cells: Differentiation and Functions. Clin Dev Immunol (2012) 2012:925135. doi: 10.1155/2012/925135 22474485 PMC3312336

[274] Williams MA Tyznik AJ Bevan MJ. Interleukin-2 Signals During Priming are Required for Secondary Expansion of CD8+ Memory T Cells. Nature (2006) 441(7095):890–3. doi: 10.1038/nature04790 PMC2776073 16778891

[275] Oberhardt V Luxenburger H Kemming J Schulien I Ciminski K Giese S. Rapid and Stable Mobilization of CD8+ T Cells by SARS-CoV-2 mRNA Vaccine. Nature (2021) 597(7875):268–73. doi: 10.1038/s41586-021-03841-4 PMC8426185 34320609

[276] Baden LR El Sahly HM Essink B Kotloff K Frey S Novak R. Efficacy and Safety of the mRNA-1273 SARS-CoV-2 Vaccine. N Engl J Med (2021) 384(5):403–16. doi: 10.1056/NEJMoa2035389 PMC7787219 33378609

[277] Lustig Y Nemet I Kliker L Zuckerman N Yishai R Alroy-Preis S. Neutralizing Response Against Variants After SARS-CoV-2 Infection and One Dose of BNT162b2. N Engl J Med (2021) 384(25):2453–4. doi: 10.1056/NEJMc2104036 PMC8063887 33826815

[278] Goel RR Painter MM Apostolidis SA Mathew D Meng W Rosenfeld AM. mRNA Vaccines Induce Durable Immune Memory to SARS-CoV-2 and Variants of Concern. Sci (2021) 374(6572):abm0829. doi: 10.1126/science.abm0829 PMC9284784 34648302

[279] Collier A-RY Brown CM Mcmahan K Yu J Liu J Jacob-Dolan C. Immune Responses in Fully Vaccinated Individuals Following Breakthrough Infection With the SARS-CoV-2 Delta Variant in Provincetown, Massachusetts. medRxiv (2021) 20:2021.10.18.21265113. doi: 10.1101/2021.10.18.21265113

[280] Tarke A Sidney J Methot N Yu ED Zhang Y Dan JM. Impact of SARS-CoV-2 Variants on the Total CD4+ and CD8+ T Cell Reactivity in Infected or Vaccinated Individuals. Cell Rep Med (2021) 2(7):100355. doi: 10.1016/j.xcrm.2021.100355 34230917 PMC8249675

[281] Tarke A Coelho CH Zhang Z Dan JM Yu ED Methot N. SARS-CoV-2 Vaccination Induces Immunological T Cell Memory Able to Cross-Recognize Variants From Alpha to Omicron. Cell (2022) 185(5):847–59.e11. doi: 10.1101/2021.12.28.474333 35139340 PMC8784649

[282] Gao Y Cai C Grifoni A Müller TR Niessl J Olofsson A. Ancestral SARS-CoV-2-Specific T Cells Cross-Recognize the Omicron Variant. Nat Med (2022) 28:472–6. doi: 10.1038/s41591-022-01700-x PMC8938268 35042228

[283] Geers D Shamier MC Bogers S den Hartog G Gommers L Nieuwkoop NN. SARS-CoV-2 Variants of Concern Partially Escape Humoral But Not T-Cell Responses in COVID-19 Convalescent Donors and Vaccinees. Sci Immunol (2021) 6(59):eabj1750. doi: 10.1126/sciimmunol.abj1750 34035118 PMC9268159

[284] Keeton R Richardson SI Moyo-Gwete T Hermanus T Tincho MB Benede N. Prior Infection With SARS-CoV-2 Boosts and Broadens Ad26.COV2.S Immunogenicity in a Variant-Dependent Manner. Cell Host Microbe (2021) 29(11):1611–19.e5. doi: 10.1016/j.chom.2021.10.003 34688376 PMC8511649

[285] Melo-González F Soto JA González LA Fernández J Duarte LF Schultz BM. Recognition of Variants of Concern by Antibodies and T Cells Induced by a SARS-CoV-2 Inactivated Vaccine. Front Immunol (2021) 12. doi: 10.3389/fimmu.2021.747830 PMC8630786 34858404

[286] Riou C Keeton R Moyo-Gwete T Hermanus T Kgagudi P Baguma R. Escape From Recognition of SARS-CoV-2 Variant Spike Epitopes But Overall Preservation of T Cell Immunity. Sci Transl Med (2022) 14(631):eabj6824. doi: 10.1126/scitranslmed.abj6824 34931886 PMC9434381

[287] Zimmermann P Curtis N. Factors That Influence the Immune Response to Vaccination. Clin Microbiol Rev (2019) 32(2):e00084-18. doi: 10.1128/CMR.00084-18 30867162 PMC6431125

[288] Collier DA Ferreira IATM Kotagiri P Datir RP Lim EY Touizer E. Age-Related Immune Response Heterogeneity to SARS-CoV-2 Vaccine BNT162b2. Nature (2021) 596(7872):417–22. doi: 10.1038/s41586-021-03739-1 PMC8373615 34192737

[289] Witkowski W Gerlo S De Smet E Wejda M Acar D Callens S. Humoral and Cellular Responses to COVID-19 Vaccination Indicate the Need for Post-Vaccination Testing in Frail Population. Vaccines (2022) 10(2):260. doi: 10.3390/vaccines10020260 35214717 PMC8875521

[290] Van Praet JT Vandecasteele S De Roo A Vynck M De Vriese AS Reynders M. Dynamics of the Cellular and Humoral Immune Response After BNT162b2 Messenger Ribonucleic Acid Coronavirus Disease 2019 (COVID-19) Vaccination in COVID-19-Naive Nursing Home Residents. J Infect Dis (2021) 224(10):1690–3. doi: 10.1093/infdis/jiab458 34514509

